# Is there a place for psychedelics in sports practice?

**DOI:** 10.1017/neu.2025.13

**Published:** 2025-03-21

**Authors:** Marina A.M. Portes, Isabel Werle, Leandro J. Bertoglio

**Affiliations:** Department of Pharmacology, Federal University of Santa Catarina, Florianopolis, Santa Catarina, Brazil

**Keywords:** Resilience, Serotonin, Neuroplasticity, Inflammation, Pain, Ecstasy, Ketamine, Cannabis

## Abstract

Growing evidence suggests that psychedelic-assisted therapies can alleviate depression, anxiety, posttraumatic stress, and substance use disorder, offering relatively safe profiles, enhanced efficacy, and lasting effects after a few applications. Athletes often experience high levels of stress and pressure, making them susceptible to these psychiatric conditions. However, the effects of psychedelic substances on athletic performance remain largely unknown. Before potential acceptance, evaluating their impact on physical and physiological measures beyond mental health outcomes is crucial. Here, we aim to explore this topic and highlight research directions to advance our understanding. Preclinical studies suggest that psilocybin/psilocin, lysergic acid diethylamide (LSD), *N*,*N*-dimethyltryptamine (DMT), and ayahuasca possess anti-inflammatory and anti-nociceptive properties. Studies investigating the effects of classical psychedelics or 3,4-methylenedioxymethamphetamine (MDMA) on factors such as muscle strength, motor coordination, locomotion, endurance, fluid and electrolyte balance, hormonal regulation, and metabolism are still scarce. While adhering to regulatory frameworks, further research in animal models, athletes, and non-athletes is needed to address these gaps, compare psychedelics with commonly used psychoactive drugs, and explore the potential prophylactic and regenerative benefits of specific interventions.

## Graphical abstract

**Figure:**
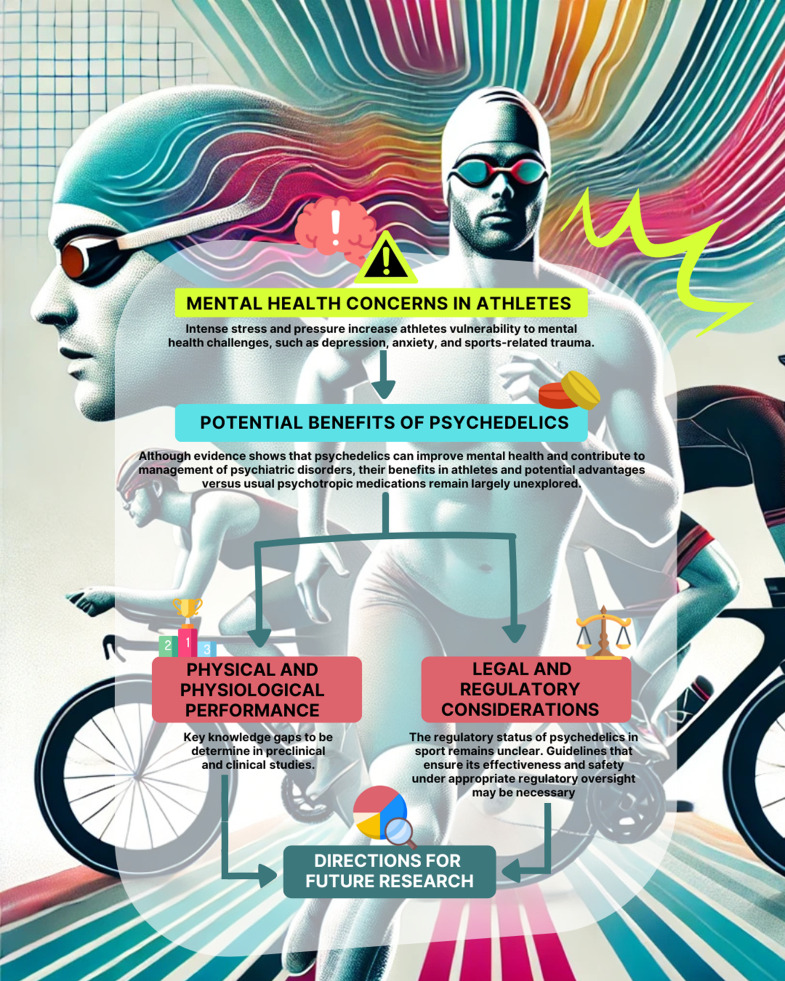

>Highlights

- Athletes frequently experience intense stress and pressure, increasing their vulnerability to mental health challenges such as depression, anxiety, and sports-related trauma.
- While psychedelics hold the potential for alleviating these issues, their impact on physical and physiological performance in athletes remains largely unexplored.
- This perspective explores the effects of psilocybin, LSD, DMT, and MDMA on mental and physical health, identifying key knowledge gaps and proposing future research directions using rodent models relevant to athletic populations.

Summations

- Psychedelic-assisted therapies are increasingly known for their potential to mitigate symptoms of various psychiatric conditions.
- Psychedelics may offer intriguing possibilities for enhancing resilience, aiding recovery, and treating sports-related trauma.
- As scientific understanding evolves, specific psychedelic substances could emerge as complementary tools in sports medicine.

Perspectives

- Research on the effects of psychedelics on physical performance and physiological parameters is still limited in rodents and humans.
- Permitting specific psychedelics in sports competitions will require a strong scientific foundation and a revision of anti-doping regulations.
- Establishing proper guidelines, dosages, and usage contexts will be crucial to ensure their responsible application.

## Introduction

Psychedelics are currently defined as psychoactive substances that alter sensory perception, thought patterns, mood, and emotional experiences, affecting numerous cognitive processes (Nichols, 2016). They induce profound changes in consciousness, including visual and auditory hallucinations, an altered perception of time, and a heightened sense of interconnectedness – effects often attributed to serotonin (5-HT) transmission in the brain (Osmond, 1957; Wittmann *et al*., 2007; Nichols, 2016; Yanakieva *et al*., 2019; Vollenweider & Preller, 2020).

Psychedelic compounds can be classified according to their chemical structure or mechanism of action (Mitchell & Anderson, 2024). Serotonergic psychedelics fall into two main structural categories, characterised by modifications in the tryptamine or the phenethylamine group (Mendes *et al*., 2022). The first category includes psilocybin (psilocin is the active metabolite) found in certain mushrooms, *N*,*N*-dimethyltryptamine (**DMT**) present in ayahuasca^1^, and 5-methoxy-*N*,*N*-DMT (**5-MeO-DMT**) derived from certain toad species. The second comprises mescaline, the primary psychoactive component of peyote cacti, and synthetic compounds such as (±)-2,5-dimethoxy-4-iodoamphetamine hydrochloride (**DOI**). Lysergic acid diethylamide (**LSD**) is an ergoline-derived compound.

Classical psychedelics (psilocybin, DMT, 5-MeO-DMT, mescaline, and LSD) act as partial or full agonists at 5-HT receptors, primarily 5-HT_2A_, 5-HT_1A_, and 5-HT_2C_ (Werle & Bertoglio, 2024). In contrast, compounds like DOI are relatively more selective agonists at 5-HT_2A_ receptors (Werle & Bertoglio, 2024). Some substances associated with psychedelics act through distinct mechanisms. For example, 3,4-methylenedioxymethamphetamine (**MDMA**) produces psychoactive effects primarily by releasing monoamines (5-HT, noradrenaline, and dopamine) and inhibiting their reuptake; ketamine is a glutamate N-methyl-D-aspartate (**NMDA**) receptor antagonist; and ibogaine (noribogaine is the active metabolite) interacts with multiple molecular targets, including 5-HT_2A_ receptors, NMDA receptors, and monoamine transporters (Johnson *et al*., 2019; Mendes *et al*., 2022).

Activating 5-HT_2A_ receptors, primarily those expressed in the apical dendrites of human layer V cortical pyramidal neurones, is essential for the perceptual effects of psychedelic experiences (Madsen *et al*., 2019). The canonical 5-HT_2A_ receptor signalling pathway involves the activation of G_αq/11_-proteins and subsequent activation of the enzyme phospholipase C, leading to hydrolysis of phosphatidylinositol-4,5-bisphosphate and the release of inositol triphosphate and diacylglycerol. 5-HT_2A_ receptors also interact with arrestins, recruiting intracellular signalling pathways dependent on these proteins (Kim *et al*., 2020; McClure-Begley & Roth, 2022; Wallach *et al*., 2023). The psychedelic potential of some phenethylamine analogues is associated with the efficacy of 5-HT_2A_-Gq but not 5-HT_2A_-β-arrestin-2 recruitment (Wallach *et al*., 2023).

Increasing evidence suggests that 5-HT_2A_ receptor agonism does not fully explain the pharmacological effects of psychedelics (Inserra *et al*., 2021; Mendes *et al*., 2022; Werle *et al*., 2024). Their action also involves the brain activation of other serotonergic and dopaminergic receptor subtypes (Werle & Bertoglio, 2024), tropomyosin receptor kinase B (**TrkB**) (Moliner *et al*., 2023; Shafiee *et al*., 2024), ionotropic glutamate receptor interactions (Heresco-Levy & Lerer, 2024), neurotransmitters release (White *et al*., 1996; Mason *et al*., 2020), increased expression of the brain-derived neurotrophic factor (**BDNF**; He *et al*., 2005; de Almeida *et al*., 2019; Marton *et al*., 2019; Hutten *et al*., 2020b; Shafiee *et al*., 2024), and epigenetic changes (Inserra *et al*., 2024). How psychedelics influence the abovementioned targets/mechanisms is complex, with each substance exhibiting particular features (Ray, 2010; Cameron *et al*., 2023).

The ability to induce adaptive structural and functional changes in the brain is a common feature of psychedelics shown in both preclinical and clinical studies (Ly *et al*., 2018; Lukasiewicz *et al*., 2021; de Vos *et al*., 2021; Liao *et al*., 2025). These substances induce neuroplasticity in response to intrinsic or extrinsic stimuli, modifying the strength and efficacy of synaptic transmission (Calder & Hasler, 2023). The cascade of cellular and molecular events implicated includes transmembrane and cytosolic receptor activation (Preller *et al*., 2018; Moliner *et al*., 2023; Vargas *et al*., 2023), recruitment of secondary messengers and proteins (Olson, 2022), changes in the number or complexity of dendritic spines (Ly *et al*., 2018; Shao *et al*., 2021), generation of new neurones (Lima da Cruz *et al*., 2018; Morales-Garcia *et al*., 2020), among others. Moreover, psychedelics can induce varying effects on functional connectivity across brain networks, such as decreased connectivity within the default mode network associated with self-referential thoughts and the sense of ego (Carhart-Harris *et al*., 2012; Palhano-Fontes *et al*., 2015; Carhart-Harris, Muthukumaraswamy, *et al*., 2016; Preller *et al*., 2020; Daws *et al*., 2022; Siegel *et al*., 2024). These changes may shift rigid thought patterns into more integrated and flexible thinking, potentially leading individuals to new insights and perspectives on life experiences. The altered states of consciousness induced by psychedelics may also affect emotional processing and facilitate coping with difficult emotions or traumatic experiences, leading to improved mental health outcomes and even therapeutic benefits (Kraehenmann *et al*., 2015; Barrett *et al*., 2020; Mertens *et al*., 2020; Arruda Sanchez *et al*., 2024; Stoliker *et al*., 2024; Melani *et al*., 2025).

Psychedelics were categorised as Schedule I substances under the Controlled Substances Act by the United States Drug Enforcement Administration in the 1970s, a decision mirrored by regulatory agencies in other countries. This classification significantly restricted academic and clinical research. However, over the past ten years, scientific and medical interest has been resurgent in exploring the pharmacological effects of these substances. As described in the following two sections, studies indicate that psychedelics have a relatively good safety profile, produce rapid benefits, and exert enduring effects after just a few doses (Riba *et al*., 2003; Palhano-Fontes *et al*., 2019; Mitchell *et al*., 2021; Gukasyan *et al*., 2022; Rhee *et al*., 2023; Dos Santos & Hallak, 2024; Hinkle *et al*., 2024). As a result, their therapeutic potential has been explored, presenting a promising approach for treating various psychiatric disorders, as detailed in Section 3 and Table 1. The subsequent sections will evaluate the potential applications of psychedelics for maintaining and improving mental wellness in athletes, their effects on physical and physiological parameters pertinent to athletic performance, and the relevant legal and regulatory frameworks.

**Table 1.** Effects of single or repeated administration of psilocybin, LSD, ayahuasca, DMT, or MDMA on the mental health of individuals diagnosed with selected psychiatric disorders

Treatment		Participants			Study features					
Psychedelic substance	Dose, administration route, and posology	Number, age in years, sex	Ethnicity	Clinical condition	Design	Psychotherapeutic support	Effect on symptoms	Benefit duration	Report of severe adverse event(s)	Reference
**Affective disorders (mainly depression)**										
Psilocybin	10 and 25 mg (1^st^ and 2^nd^ session), oral, one week apart	12, 30–64, ♂♀	Mostly White or Caucasian (75%)	Moderate-to-severe MDD	Open-label	Yes	↓	At least 3 months post-treatment	No	Carhart-Harris *et al*., 2016b
Psilocybin	1 or 3 mg/70 kg (1^st^ session) and 22 or 30 mg/70 kg (2^nd^ session), oral, 5 weeks apart	51, 56.3 ± 1.4 (mean ± SEM), ♂♀	Mostly White or Caucasian (94%)	Cancer-related anxiety and depression	R, D-B, cross-over	Yes	↓	At least 6 months post-treatment	No	Griffiths *et al*., 2016
Psilocybin	0.3 mg/kg, oral, single administration	29, 56 ± 13 (mean ± SD), ♂♀	Mostly White or Caucasian (90%)	Cancer-related anxiety and depression	R, D-B, P-C, cross-over	Yes	↓	At least 6 months post-treatment	No	Ross *et al*., 2016
Ayahuasca	120–200 ml (adjusted to contain 96–160 mg of DMT, and 25–42 mg of harmine), oral, single administration	17, 43 ± 12 (mean ± SD), ♂♀	n.d.	Moderate-to-severe TR-MDD	Open-label	n.d.	↓	At least 21 days post-treatment	No	Sanches *et al*., 2016
Psilocybin	10 and 25 mg (1^st^ and 2^nd^ session), oral, 1 week apart	19, 42.8 (mean), ♂♀	n.d.	TR-MDD	Open-label	n.d.	↓	At least 5 weeks post-treatment	n.d.	Carhart-Harris *et al*., 2017
Psilocybin	10 and 25 mg (1^st^ and 2^nd^ session), oral, one week apart	20, 44 ± 11 (mean ± SD), ♂♀	Mostly White or Caucasian (55%)	Severe MDD	Open-label	Yes	↓	At least 6 months post-treatment	No	Carhart-Harris *et al*., 2018
Ayahuasca	1 ml/kg (adjusted to contain 0.36 mg/kg of DMT), oral, single administration	71, 42 ± 12 (mean ± SD), ♂♀	n.d.	TR-MDD	R, D-B, P-C	n.d.	n.d.	n.d.	n.d.	Galvão *et al*., 2018
Psilocybin	10 and 25 mg (1^st^ and 2^nd^ session), oral, 1 week apart	20, 45 ± 11 (mean ± SD), ♂♀	n.d.	Moderate-to-severe TR-MDD	Open-label	Yes	↓	At least 3–5 weeks post-treatment	n.d.	Roseman *et al*., 2018
Ayahuasca	1 ml/kg, oral, single administration	83, 42 ± 11 (mean ± SD), ♂♀	n.d.	TR-MDD	R, D-B, P-C	n.d.	n.d.	n.d.	n.d.	de Almeida *et al*., 2019
Ayahuasca	1 ml/kg (adjusted to contain 0.36 mg/kg of DMT), oral, single administration	29, 40 ± 11 (mean ± SD), ♂♀	Mostly White or Caucasian (64%)	Moderate-to-severe TR-MDD	R, D-B, P-C	n.d.	↓	At least 7 days post-treatment	No	Palhano-Fontes *et al*., 2019
Psilocybin	10 and 25 mg (1^st^ and 2^nd^ session), oral, 1 week apart	19, 45 ± 11 (mean ± SD), ♂♀	n.d.	Moderate-to-severe TR-MDD	Open-label	Yes	↓	At least 5 weeks post-treatment	n.d.	Mertens *et al*., 2020
Psilocybin	25 mg, oral, two admin. (one every 3 weeks)	59, 43 ± 12 (mean ± SD), ♂♀	Mostly White or Caucasian (93%)	Moderate-to-severe MDD	R, D-B, controlled	Yes	↓	At least 6 weeks post-treatment	No	Carhart-Harris *et al*., 2021
Psilocybin	20 and 30 mg/70 kg (1^st^ and 2^nd^ session), oral, two admin. (1.6 weeks apart)	24, 40 ± 12 (mean ± SD), ♂♀	n.d.	Moderate-to-severe MDD	R	Yes	↓	At least 8 weeks post-treatment	No	Davis *et al*., 2021
Ayahuasca	2.2 ml/kg (adjusted to contain 0.8 mg/ml of DMT, 0.21 mg/ml of harmine, and no harmaline)	17, 43 ± 12 (mean ± SD), ♂♀	n.d.	TR-MDD	Open-label	No	↓	At least 21 days post-treatment	n.d.	Zeifman *et al*., 2021
DMT	0.1 or 0.3 mg/kg, i.v., two administrations (one every 48 h)	10, 24-59, ♂♀	Mostly White or Caucasian (70%)	TR-MDD	Open-label, fixed-order, dose-escalation	No	↓	At least 1 day post-treatment	Asymptomatic bradycardia and hypotension (n = 1)	D’Souza *et al*., 2022
Psilocybin	1, 10, or 25 mg, oral, single administration	233, 40 ± 12 (mean ± SD), ♂♀	Mostly White or Caucasian (92.3%)	TR-MDD	R, D-B, parallel-group	Yes	↔ (1 and 10 mg) ↓ (25 mg)	At least 3 weeks post-treatment	Suicidal behaviour (n = 3), codeine withdrawal syndrome (n = 1), and adjustment disorder with anxiety and depressed mood (n = 1)	Goodwin *et al*., 2022
Psilocybin	20 or 30 mg/70 kg, oral, two administrations (one every 2 weeks)	24, 40 ± 12 (mean ± SD), ♂♀	Mostly White or Caucasian (92%)	Moderate-to-severe MDD	R, waiting-list controlled study	Yes	↓	At least 12 months post-treatment	No	Gukasyan *et al*., 2022
Ayahuasca	50 ml (containing 3, 4, or 5 g of *Peganum harmala*, and 9, 15, or 16.67 g of *Mimosa hostilis*), oral, two administrations (one every 2.5 h)	20, 35 ± 10 (mean ± SD), ♂♀	n.d.	Chronic MDD (> 2 years)	Longitudinal observational study	n.d.	↓	At least 12 months post-treatment	n.d.	van Oorsouw *et al*., 2022
Psilocybin	25 mg, oral, single administration	19, 42 ± 11 (mean ± SD), ♂♀	Mostly White or Caucasian (78.9%)	TR-MDD	Open-label	Yes	↓	At least 3 weeks post-treatment	No	Goodwin *et al*., 2023
Psilocybin	0.3 mg/kg, oral, single administration	25, 43 ± 14 (mean ± SD), ♂♀	n.d.	TR-MDD	D-B, P-C, within-subject study	Yes	↓	At least 2 weeks post-treatment	n.d.	Skosnik *et al*., 2023
Psilocybin	0.3 mg/kg, oral, single administration	19, 43 ± 14 (mean ± SD), ♂♀	Mostly White or Caucasian (84.2%)	TR-MDD	P-C, within-subject, fixed-order	Yes	↓	At least 2 months post-treatment	No	Sloshower *et al*., 2023
Psilocybin	1 or 25 mg, oral, two administrations (one every 3 weeks)	59, 43 ± 10 (mean ± SD), ♂♀	Mostly White or Caucasian (93.3%)	MDD	R, D-B	Yes	↔ (1 mg) ↓ (25 mg)	At least 6 weeks post-treatment	n.d.	Zeifman *et al*., 2023
Psilocybin	25 mg, oral, single administration	15, 38 ± 12 (mean ± SD), ♂♀	Mostly White or Caucasian (80%)	Bipolar disorder depression	Open-label	Yes	↓	At least 12 weeks post-treatment	No	Aaronson *et al*., 2024
Psilocybin	1, 10, or 25 mg, oral, single administration	11, 45.5 (mean), ♂♀	n.d.	TR-MDD	R, D-B	Yes	n.d.	At least 12 weeks post-treatment	n.d.	Breeksema *et al*., 2024
Psilocybin	1 or 25 mg, oral, two administrations (one every 3 weeks)	59, n.d., n.d.	n.d.	Moderate-to-severe MDD	R, D-B, two-arm, controlled trial	n.d.	↔ (1 mg) ↓ (25 mg)	At least 2 weeks post-treatment	n.d.	Peill *et al*., 2024
Psilocybin	0.3 mg/kg, oral, single administration	15, 43 ± 14 (mean ± SD), ♂♀	Mostly White or Caucasian (84.2%)	Moderate-to-severe MDD	P-C, within-subject, fixed-order	Yes	↓	At least 16 months post-treatment	n.d.	Sloshower *et al*., 2024
**Posttraumatic stress disorder**										
MDMA	125 mg, oral, two administrations (one per month)	20, 21–70, ♂♀	100% White or Caucasian	PTSD	R, D-B, P-C	Yes	↓	At least 2 months post-treatment	No	Mithoefer *et al*., 2011
MDMA	125–187.5 mg, oral, two administrations (one per month)	19, n.d., n.d.	100% White or Caucasian	PTSD	Prospective long-term follow-up	Yes	↓	17–74 months post-treatment	n.d.	Mithoefer *et al*., 2013
MDMA	37.5 or 187.5 mg, oral, three administrations (interval n.d.)	12, 41 ± 11 (mean ± SD), ♂♀	n.d.	PTSD	R, D-B, active- P-C	Yea	↔ (37.5 mg) ↓ (187.5 mg)	At least 12 months post-treatment	No	Oehen *et al*., 2013
MDMA	30, 75, or 125 mg, oral, two administrations (one every 3–5 weeks)	26, 37 ± 10 (mean ± SD), ♂♀	Mostly White or Caucasian (85%)	PTSD	R, D-B	Yes	↔ (30 mg) ↓ (75 and 125 mg)	At least 12 weeks post-treatment	No	Mithoefer *et al*., 2018
MDMA	40, 100, or 125 mg, oral, two administrations (one per month)	28, 42 ± 13 (mean ± SD), ♂♀	n.d.	PTSD	R, D-B	Yes	↔ (40 mg) ↓ (100 or 125 mg)	At least 2 months post-treatment	No	Ot’alora *et al*., 2018
MDMA	75 to 125 mg, oral, 2 or 3 administrations (one every 3–5 weeks)	105, 40 ± 11 (mean ± SD), ♂♀	Mostly White or Caucasian (87.6%)	PTSD	R, D-B, P-C	Yes	↓	At least 1–2 months after two experimental sessions	No	Mithoefer *et al*., 2019
MDMA	75–187.5 mg, oral, two administrations (one every 3–5 weeks)	60, 41 ± 12 (mean ± SD), ♂♀	Mostly White or Caucasian (84.4%)	PTSD	R and two-blinded	Yes	↓	At least 1–2 months post-treatment	n.d.	Gorman *et al*., 2020
MDMA	75–125 mg, oral, 2 or 3 administrations (one every 2–3 weeks)	107, 40 ± 11 (mean ± SD), ♂♀	Mostly White or Caucasian (89.7%)	PTSD	Blinded study segment, open-label cross-over, long-term follow-up	Yes	↓	At least 12 months post-treatment	n.d.	Jerome *et al*., 2020
MDMA	125 mg, oral, two administrations (one every 2–4 weeks)	18, 55 ± 8 (mean ± SD), ♂♀	Mostly White or Caucasian (60%)	Psychiatric medical history diagnosis of anxiety, major depression, PTSD, or/and insomnia	R, D-B, P-C	Yes	↓	At least 12 months post-treatment	n.d.	Wolfson *et al*., 2020
MDMA	75–187.5 mg, oral, three administrations (one per month)	3, 40.3 (mean), 1 ♂ and 2 ♀	One Afro-Brazilian and two Caucasian	PTSD	Open-label	Yes	↓	At least 2 months post-treatment	n.d.	Jardim *et al*., 2021
MDMA	80–180 mg, oral, three administrations (one every 4 weeks)	90, 41 ± 12 (mean ± SD), ♂♀	Mostly White or Caucasian (76.7%)	Severe PTSD	R, D-B, P-C	Yes	↓	At least 18 weeks post-treatment	n.d.	Mitchell *et al*., 2021
MDMA	75–187.5 mg, oral, 2 or 3 administrations (one every 3–5 weeks)	63, 41 ± 11 (mean ± SD), ♂♀	Mostly White or Caucasian (85.7%)	PTSD	R, D-B, P-C	Yes	↓	At least 2 months post-treatment	n.d.	Ponte *et al*., 2021
MDMA	80–240 mg, oral, three administrations (one every 4 weeks)	89, 41 ± 12 (mean ± SD), ♂♀	Mostly White or Caucasian (78.16%)	PTSD	R, D-B, P-C pivotal trial	Yes	↓	n.d.	n.d.	Brewerton *et al*., 2022
MDMA	80–190 mg, oral, three administrations (interval n.d.)	127, 37.9 (mean), ♂♀	Mostly White or Caucasian (81.9%)	PTSD	Two Phase 2 open-label trials and a Phase 3 R, blinded P-C trial	n.d.	↓	n.d.	No	Ching *et al*., 2022
MDMA	120–180 mg, oral, three administrations (one every 3–4 weeks)	82, 41 ± 12 (mean ± SD), ♂♀	Mostly White or Caucasian (80.25%)	PTSD plus SUD	R, three-blind, P-C	Yes	↓	At least 6 months post-treatment	n.d.	Nicholas *et al*., 2022
MDMA	120–180 mg, oral, three administrations (one per month)	104, 38 ± 11 (mean ± SD), ♂♀	Mostly White or Caucasian (69.8%)	Moderate-to-severe PTSD	R, D-B, P-C	Yes	↓	At least 18 weeks post-treatment	No	Mitchell *et al*., 2023
MDMA	30, 75, or 125 mg, oral, two administrations	9, 41 ± 11 (mean ± SD), ♂♀	Mostly White or Caucasian (89%)	PTSD	R, D-B	Yes	↓	At least 2 months post-treatment	n.d.	Singleton *et al*., 2023
MDMA	120–180 mg, oral, three administrations (one every 4 weeks)	90, 41 ± 12 (mean ± SD), ♂♀	Mostly White or Caucasian (80.3%)	PTSD	R, D-B, P-C, multi-site	Yes	↓	At least 2 months post-treatment	n.d.	van der Kolk *et al*., 2024
**Anxiety disorders**										
Psilocybin	0.2 mg/kg, oral, single administration	8, 36–58, ♂♀	n.d.	Advanced-stage cancer and anxiety	R, D-B, P-C	Yes	↓	At least 3–6 months post-treatment	No	Grob *et al*., 2011
LSD	20 or 200 μg, oral, two administrations (one every 2–3 weeks)	11, 52 ± 9 (mean ± SD), ♂♀	n.d.	Anxiety associated with life-threatening diseases	R, D-B, active P-C	Yes	↓	At least 12 months post-treatment	No	Gasser *et al*., 2014
LSD	20 or 200 μg, oral, two administrations (one every 4–6 weeks)	10, 51.1 (mean), ♂♀	n.d.	Anxiety associated with life-threatening	R, D-B, active P-C study followed by cross-over study	Yes	↓	At least 12 months post-treatment	No	Gasser *et al*., 2015
Psilocybin	1 or 3 mg/70 kg (1^st^ session)and 22 or 30 mg/70 kg (2^nd^ session), oral, 5 weeks apart	51, 56 ± 1 (mean ± SEM), ♂♀	Mostly White or Caucasian (94%)	Cancer-related anxiety and depression	R, D-B, cross-over	Yes	↓	At least 6 months post-treatment	No	Griffiths *et al*., 2016
Psilocybin	0.3 mg/kg, oral, single administration	29, 56 ± 13 (mean ± SD), ♂♀	Mostly White or Caucasian (90%)	Cancer-related anxiety and depression	R, D-B, P-C, cross-over	Yes	↓	At least 6 months post-treatment	No	Ross *et al*., 2016
MDMA	15, 100, or 125 mg, oral, two admin. (one per month)	12, 31 ± 9 (mean ± SD), ♂♀	Mostly White or Caucasian (50%)	Autism with social anxiety	R, D-B, P-C	Yes	↔ (15 mg) ↓ (100 or 125 mg)	At least 6 months post-treatment	No	Danforth *et al*., 2018
Ayahuasca	2 ml/kg, oral, single administration	17, 25 (mean) years, ♂♀	n.d.	SAD	R, D-B, P-C, parallel-group	No	↓	n.d.	n.d.	dos Santos *et al*., 2021
LSD	200 μg, oral, two admin. (one every 6 weeks)	42, 45 ± 12 (mean ± SD), ♂♀	n.d.	Anxiety with and without a life-threatening illness	R, D-B, P-C, cross-over	Yes	↓	At least 16 weeks post-treatment	Acute transient anxiety (2%)	Holze *et al*., 2023
**Eating disorders**										
Psilocybin	25 mg, oral, single administration	10, 28 ± 4 (mean ± SD), ♀	Mostly White or Caucasian (90%)	Anorexia nervosa	Open-label	Yes	↓	At least 3 months post-treatment	No	Peck *et al*., 2023
Psilocybin	25 mg, oral, single administration	12, 34 ± 9 (mean ± SD), ♂♀	Mostly White or Caucasian (75%)	Moderate-to-severe non-delusional body dysmorphic disorder unresponsive to serotonin reuptake inhibitor(s)	Open-label	Yes	↓	At least 12 weeks post-treatment	No	Schneier *et al*., 2023
**Substance use disorders**										
Psilocybin	20 or 30 mg/70 kg, oral, two administrations (one every 2 weeks)	15, 51 ± 10 (mean ± SD), ♂♀	Mostly White or Caucasian (93%)	Nicotine-dependent smokers	Open-label	Yes	↓	n.d.	No	Johnson *et al*., 2014
Psilocybin	0.3 or 0.4 mg/kg, oral, two administrations (one every 8 weeks)	10, 40 ± 10 (mean ± SD), ♂♀	Native American/Alaska Native (n = 2), African American (n = 1), Hispanic (n = 4), and White non-Hispanic (n = 3)	Ethanol dependence	Open-label	Yes	↓	At least 36 weeks post-treatment	No	Bogenschutz *et al*., 2015
Psilocybin	20 or 30 mg/70 kg, oral, two administrations (one every 2 weeks)	15, 51 (mean), ♂♀	Mostly White or Caucasian (93%)	Nicotine-dependent smokers	Open-label	Yes	↓	At least 16 months post-treatment	No	Johnson *et al*., 2017
Psilocybin	25 mg/70 kg (1^st^ session)and 25 to 40 mg/70 kg (2^nd^ session), oral, 4 weeks apart	96, 46 ± 12 (mean ± SD), ♂♀	Mostly White or Caucasian (79%)	Ethanol dependence	R, D-B, controlled trial	Yes	↓	At least 36 weeks post-treatment	No	Bogenschutz *et al*., 2022
Psilocybin	25 mg/70 kg, oral, single administration	11, 49 ± 11 (mean ± SD), ♂♀	Mostly White or Caucasian (72%)	AUD	R, D-B, P-C	Yes	n.d.	n.d.	n.d.	Pagni *et al*., 2024

**Legend:** ↔ = relatively no changes; ↓ = reduction; ♂ = men; ♀ = women; AUD = alcohol use disorder; D-B = double-blind; i.v. = intravenous route; MDD = major depressive disorder; MDMA = 3,4-methylenedioxymethamphetamine; n.d. = not described; DMT = *N*,*N*-dimethyltryptamine; P-C = placebo-controlled; PTSD = posttraumatic stress disorder; R = randomised; SAD = social anxiety disorder; SD = standard deviation; SEM = standard error of mean; SUD = substance use disorder; TR-MDD = treatment-resistant major depressive disorder.

## On the safety of psychedelics

The acute toxicity of psychedelics is considered low. Reports of fatal overdoses associated with their use are rare (Haden & Woods, 2020; Darke *et al*., 2024; Thomas, 2024), with deaths primarily linked to relatively high doses (i.e. ≥ 20 times the typical dose) or the combination of psychedelics with other drugs or ethanol (Schlag *et al*., 2022; Lake & Lucas, 2024; Kopra *et al*., 2025). Clinical studies conducted in supervised settings have also demonstrated low addictive potential (Johnson *et al*., 2008; Johansen & Krebs, 2015; Johnson *et al*., 2018; Schlag *et al*., 2022; Hinkle *et al*., 2024). Compared to ethanol, opioids, cocaine, crack, amphetamines, and some psychostimulants, they have a low risk of addiction and intoxication (Johnson *et al*., 2018). Noteworthy, clinical evidence suggests that psychedelics can alleviate psychological and physiological symptoms associated with dependence on other psychoactive substances (Vamvakopoulou & Nutt, 2024; Yao *et al*., 2024).

Challenging emotional experiences (e.g. anxiety and panic attacks), sensory and spatial distortions, headache, nausea and vomiting, and elevations in heart rate and blood pressure are changes induced by psychedelics as *transient* effects observed after administering usual doses but infrequently manifest in protocols using microdoses (Nichols, 2016; Polito & Stevenson, 2019; Schlag *et al*., 2022; Wsół, 2023; Murphy *et al*., 2024; Neumann *et al*., 2024; Yerubandi *et al*., 2024) or when used in controlled settings with appropriate inclusion criteria (Rhee *et al*., 2023; Hinkle *et al*., 2024; Klaiber *et al*., 2024; Romeo *et al*., 2024; Simon *et al*., 2024; Sabé *et al.,* 2025). These relatively limited adverse reactions are associated with stimulating various 5-HT receptors (Johnson *et al*., 2008; Family *et al*., 2022; Holze *et al*., 2022). For example, the potential cardiovascular risk associated with serotonergic psychedelics is attributed to their interaction with 5-HT_1B_, 5-HT_2B_, and 5-HT_4_ receptors (Wsół, 2023). However, no associations have been established between the lifetime use of classical psychedelics and the development of cardiometabolic diseases (Simonsson *et al*., 2021).

The relationship between psychedelic use and the risk of seizures is not fully understood, as clinical studies typically exclude individuals with a history of seizures or convulsions. While psychedelics may theoretically increase the risk in predisposed individuals due to cortical 5-HT_2A_ receptor hyperstimulation, most studies suggest that these substances have a low epileptogenic potential when used in controlled settings. The risk may be elevated when psychedelics are combined with factors common in athletic environments, such as sleep deprivation, stimulant use, or high-stress conditions, particularly in susceptible individuals. However, further investigation is needed to understand better the underlying mechanisms and associated risk factors (Freidel *et al*., 2024; Lewis *et al*., 2024; Soto-Angona *et al*., 2024). Based on this, caution is advised for athletes with a history of seizures and those using medications (e.g. bupropion) or supplements (e.g. high-dose caffeine) that may lower the seizure threshold.

The use of psychedelics, particularly MDMA, has been associated with an increased risk of hyponatremia in humans, primarily due to increased antidiuretic hormone (also known as vasopressin) release from the posterior pituitary and excessive fluid intake, leading to water retention and sodium dilution (Atila *et al*., 2024). This mechanism is attributed to MDMA’s elevation of hypothalamic 5-HT and dopamine levels, stimulating vasopressin release and promoting water retention via vasopressin-2 receptors in the kidneys. Excessive water intake, driven by hyperthermia, dry mouth, and stimulant effects in physically demanding or hot environments, may exacerbate sodium dilution. Although this effect is self-limiting and observed mainly acutely, this condition may be particularly concerning for endurance athletes. Temporary hyponatremia outside of competition may contribute to longer-term consequences, potentially predisposing them to a higher risk of injuries or reduced performance in subsequent training or competitions.

Evidence from both rodent and human studies has demonstrated an association between MDMA use and an increased risk of hyperthermia and rhabdomyolysis. MDMA-treated rodents exhibited significant increases in body temperature, sustained muscle contraction, and muscle damage resembling rhabdomyolysis. These effects were related to increases in neurotransmitters, primarily 5-HT and dopamine, and activation of the sympathetic nervous system (Sprague *et al*., 2004; Duarte *et al*., 2005; Rusyniak *et al*., 2005; Sprague *et al*., 2005; de Bragança *et al*., 2017). In humans, clinical and observational studies have reported similar effects, especially in intoxication or recreational settings involving prolonged physical activity, crowded environments, and inadequate thermoregulation (Screaton *et al*., 1992; Lehmann *et al*., 1995; Halachanova *et al*., 2001; Sue *et al*., 2002; Vanden Eede *et al*., 2012; Doyle *et al*., 2020). This could be relevant for endurance athletes if their MDMA use and physical exercise are not adequately spaced apart, as MDMA-induced hyperthermia and rhabdomyolysis can be exacerbated by the physiological demands of prolonged exertion, increasing the risk of severe complications and impairing athletic performance. Although the cited articles did not assess athletes under the acute effects of psychedelic substances, their findings indirectly underscore the importance of understanding the risks associated with MDMA use in physically demanding contexts, as well as the need for proper monitoring of signs and symptoms.

Evidence indicates that 5-HT is a key neuromodulator of locomotor activity (Bacqué-Cazenave *et al*., 2020; Flaive *et al*., 2020). As reviewed by Werle and Bertoglio (2024), published studies have demonstrated the biphasic effects of psychedelic substances on locomotion. In the open-field test, rats and mice exhibit either hyperlocomotion or hypolocomotion, depending on the dose. These effects are mediated by mechanisms involving the activation of 5-HT_1A_, 5-HT_2C_, and 5-HT_2A_ receptors (in the case of MDMA, they also involve the release of 5-HT and dopamine). Each substance has its particularities, although hypolocomotor effects (suggestive of sedation) generally predominate at moderate to high doses (Werle & Bertoglio, 2024). While it is unlikely and strongly discouraged for individuals to participate in sports while under the acute influence of psychedelics, it is worth noting that rodent studies suggest psilocybin, LSD, DMT, ayahuasca, and MDMA can influence locomotor activity.

## Psychedelics and mental health

Psychedelics can provide significant benefits across multiple domains of mental health and well-being in healthy individuals (Lebedev *et al*., 2016; Schmid & Liechti, 2018; Hutten *et al*., 2020a; Perkins *et al*., 2022). Of particular relevance to athletes are several potential effects, including reduced pain (Ramaekers *et al*., 2021; Askey *et al*., 2024; Strand *et al*., 2025) and improvements in sleep (Allen *et al*., 2024). Additionally, psychedelics may enhance stress management by reducing anxiety levels and promoting greater emotional resilience (Griffiths *et al*., 2011; Arruda Sanchez *et al*., 2024).

The growing interest and acceptance of psychedelic substances have driven clinical trials, advancing our understanding of their potential benefits (Nichols, 2016; Reiff *et al*., 2020; Nutt & Carhart-Harris, 2021; McClure-Begley & Roth, 2022). Their contribution to alleviating symptoms of depression, anxiety, posttraumatic stress disorder (PTSD), eating disorders, and substance use disorders has been documented (Reiff *et al*., 2020; Barber & Aaronson, 2022; Brewerton *et al*., 2022; Cavarra *et al*., 2022; Cuerva *et al*., 2024; Dos Santos & Hallak, 2024; Doss *et al*., 2024; Zaretsky *et al*., 2024). Table 1 presents the details and primary findings of human studies examining the effects of psilocybin, LSD, DMT, ayahuasca, and MDMA on the mental health of individuals diagnosed with the aforementioned psychiatric conditions. Noteworthy, the association of psychedelics with psychotherapeutic support (i.e. psychedelic-assisted psychotherapy) has been shown to improve the integration of psychedelic experiences (Luoma *et al*., 2020).

Some of the studies reviewed (Table 1) also report that these substances are associated with significant and long-lasting symptom reduction, with therapeutic effects persisting for weeks or months following only a few administrations, even in patients resistant to typical pharmacological treatment. Psychedelics have also presented a favourable safety profile, as indicated by the relatively low incidence of severe adverse reactions when administered under controlled clinical conditions. Such features may be particularly relevant for health care in athletes, who often endure high levels of physical and mental stress and are vulnerable to various psychiatric disorders (Edwards, 2024). Hypothetically, psychedelic therapy could serve as a valuable tool for enhancing well-being in this population with minimal risk of impairing performance.

However, it is essential to address the methodological limitations of the studies published to date, as well as the gaps that still need to be clarified to enable a responsible application of psychedelic therapies in clinical practice. Some reviewed studies included small sample sizes and lacked double-blind methodologies or inactive placebos, which limits the generalisability of the observed results and increases the chance of confirmation bias. Furthermore, the majority of participants were White or Caucasian, which may limit the extrapolation of findings to other ethnic groups with distinct cultural or genetic characteristics, thus impacting the representativeness of these results when psychedelics are applied on a larger scale. Another issue is the variability in study protocols (e.g. dosage, number of administrations, and intervals between treatments). Greater methodological rigour and standardisation are needed to understand better the actual clinical impact of psychedelic therapy on both the general population and athletes. Future research should also incorporate more objective evaluation methods, ideally including physiological or neurobiological measurements that can be correlated with the health status (or psychiatric disorder under investigation).

## Mental health issues in athletes

Studies indicate that the prevalence of psychiatric disorders in high-performance athletes (both amateur and professional) may be similar or even higher than in the general population, which likely arises from intense physical and emotional stressors often experienced (Gouttebarge *et al*., 2019; Reardon *et al*., 2019; Glick *et al*., 2020; Marí-Sanchis *et al*., 2022; McDonald *et al*., 2023; Smith *et al*., 2023; Thuany *et al*., 2023; Beable, 2024). Among them are the high demand for physical and sports performance, overtraining, interpersonal conflicts in competitions, the imbalance between personal life and training, injuries, and early retirement (Chang *et al*., 2020). Furthermore, due to self-pressure to demonstrate mental resilience, athletes may not report their health concerns, accept professional assistance, or adhere to treatment. Additionally, athletes may often avoid pharmacological treatment due to concerns about doping, potential adverse reactions, and the effects of medication on athletic performance (Reardon, 2016; Bomfim, 2020). As a result, a cycle of untreated suffering can develop, compromising both mental health and physical aspects. Early identification of these factors and appropriate clinical intervention are essential to ensure performance and longevity in sports practice, as well as the psychological well-being of athletes (Glick *et al*., 2012; Chang *et al*., 2020). Consequently, there is growing interest in sports research to assess the mental health of athletes such as long-distance runners, cyclists, swimmers, triathletes, and others (Berger *et al*., 2024).

Drugs currently available for the management of psychiatric disorders in athletes present significant limitations (Morris, 2015; Reardon & Creado, 2016; Tso & Pelliccia, 2022). Antidepressants and anxiolytics currently approved for clinical use are administered daily and can cause side effects that negatively affect athletic performance, such as drowsiness, changes in appetite, and weight gain (Reardon, 2016; Reardon & Creado, 2016; Edwards, 2024). In addition, individual variability in response to these medications can hinder treatment effectiveness. For example, while approximately 15% of participants in clinical trials experience a significant antidepressant effect beyond that of a placebo (Stone *et al*., 2022), around 30% of individuals diagnosed with major depressive disorder are resistant to conventional treatment, further increasing the social and economic burden of this condition (McIntyre *et al*., 2023). In this scenario, psychedelic therapy could emerge as either a complementary or an alternative for the treatment of psychiatric disorders in athletes.

## Psychedelics to maintain and improve mental health in athletes

Several clinical studies have demonstrated the efficacy of psychedelic-assisted psychotherapy (Table 1; Nichols, 2016; Reiff *et al*., 2020; Nutt & Carhart-Harris, 2021; Cavarra *et al*., 2022; Knudsen, 2023). Following approval by the Therapeutic Goods Administration in 2023, Australia became the first country to authorise and regulate the medicinal use of psilocybin and MDMA for the treatment of depression and PTSD, respectively (Nutt *et al*., 2024). Similarly, Oregon and Colorado became the first American states to legalise psilocybin, issuing official licences to specialised mental healthcare service centres for use (Korthuis *et al*., 2024).

To date, the potential of psychedelics to enhance mental health or treat psychiatric disorders in athletes remains unknown. However, considering the evidence from the general population (Table 1), several aspects of psychedelic therapy may be beneficial for these individuals (Carhart-Harris & Goodwin, 2017; Barber & Aaronson, 2022; Holze *et al*., 2024). In healthy athletes, the administration of psychedelics may offer benefits in promoting mental health and well-being, aiding in the management of psychological and emotional challenges. By enhancing resilience and emotional flexibility, psychedelic therapy could mitigate the effects of everyday stressors in high-performance sports, including intensive training routines, self-imposed demands for physical performance, and sustained competitiveness. Moreover, in athletes diagnosed with psychiatric disorders, psychedelic-assisted psychotherapy could offer some advantages over conventional treatments. Unlike daily medications, only a few sessions spaced over days to weeks are typically sufficient to promote long-term mental health benefits that are maintained over several months (Yao *et al*., 2024). Furthermore, the half-life of these substances lasts only a few hours, not producing withdrawal symptoms. Although psychedelic therapy may result in adverse reactions, they are transient and manifest mainly in the following hours after administration. Thus, potential concerns associated with impaired sports performance can be reduced, even if athletes are in training or competition periods (Reardon & Creado, 2016; Edwards, 2024). Yousefi *et al*. (2025) have meta-analysed psilocybin’s acute effects on executive functions and attention. Psilocybin increased reaction times dose-dependently without significantly affecting accuracy, suggesting an impairment in executive function that may be relevant to specific sports. However, its impact on performance is potentially less concerning, as athletes are not expected to compete while under the influence of psychedelics.

Several psychedelic substances produce prosocial effects in rodent and human studies (Dumont *et al*., 2009; Hysek *et al*., 2014; Kamilar-Britt & Bedi, 2015; Griffiths *et al*., 2018; De Gregorio *et al*., 2021; Bhatt & Weissman, 2024). While systematic research on psychedelics in sports is limited, their potential prospective effects may include improved social dynamics during training or competition, team cohesion, reduced anxiety, enhanced resilience among athletes, and sports-related mild traumatic brain injury (e.g. concussion) (VanderZwaag *et al*., 2024). However, the use of psychedelics in sports raises potential issues. Serotonergic psychedelics and related compounds produce varying effects in tests of negative social interactions, often assessing aggression, in rodents through their actions on 5-HT_2A_ and 5-HT_1A_ receptors (Odland *et al*., 2022). Future studies must establish optimal dosages, contexts, and protocols that maximise potential benefits while minimising risks.

Scientific evidence on the interactions between psychedelic substances and antidepressants, antipsychotics, anxiolytics, and mood stabilisers remains limited. However, it has been reported that psychedelics and certain psychiatric medications may share overlapping pharmacological targets, molecular pathways interactions, and hepatic metabolism via similar enzymes (Sarparast *et al*., 2022; Rhee *et al*., 2023; Halman *et al*., 2024). Consequently, drug interactions between psychedelic substances and medications already used by athletes should be considered, as they may potentiate or attenuate the actions of both substances. Therefore, adequate clinical monitoring will be essential to mitigate the risks of adverse reactions, toxicity, or inadequate management of psychiatric symptoms.

## Effects of psychedelics on physical and physiological parameters

Administration of the psychedelic substance DOI has been shown to reduce circulating levels of total cholesterol and low-density lipoprotein (**LDL**) in a high-fat diet-fed apolipoprotein E knockout mice model without affecting food intake or body weight. DOI administration was also associated with a reduction in the increased serum levels of the pro-inflammatory cytokine CXCL10 induced by high-fat diet-fed and reduced expression of pro-inflammatory marker genes in the aortic arch (Flanagan *et al*., 2019a). On the other hand, preclinical studies have shown potentially conflicting results of the psilocybin administration on metabolic parameters and body weight regulation. Although the administration of a high dose of psilocybin was associated with a modest but significant reduction in body weight, decreased consumption of the high-calorie diet, and decreased central adiposity in a rodent model of obesity (Huang *et al*., 2022), neither a single nor repeated administration of psilocybin had significant metabolic effects. It did not lower body weight or food intake in diet-induced obese mice or genetic mouse models of obesity (Fadahunsi *et al*., 2022). Moreover, increased creatine kinase, aspartate aminotransferase, and chloride have been reported in male and female mice treated with psilocybin (Shakir *et al*., 2024). Preclinical studies have shown that MDMA treatment may increase serum levels of total and LDL cholesterol, corticosterone, aspartate transaminase, alanine transaminase, or glucose in rodents (Graham *et al*., 2010; Shahraki & Irani, 2014; Golchoobian *et al*., 2017), although hypoglycaemia has also been reported (Soto-Montenegro *et al*., 2007; Golchoobian *et al*., 2017).

In addition to regulating body weight, lipid metabolism is also essential for cellular mechanisms related to inflammation and nociception/pain, and the anti-inflammatory and immunomodulatory properties of psychedelics have also been reported (Flanagan & Nichols, 2022). Lipid mediators, including arachidonic acid (AA), can be metabolised by cyclooxygenase (COX), lipoxygenase (LOX), and cytochrome P450 (CYP450) enzymes and converted to pro-inflammatory metabolites such as prostaglandins, thromboxane, leukotrienes, and hydroxyeicosatetraenoic acids. In rodents, the psychedelic bufotenine has been shown to induce an anti-nociceptive effect and promote the downregulation of inflammatory mediators from COX, LOX, CYP450, linoleic acid, docosahexaenoic acid, and other pro-inflammatory pathways (Wang *et al*., 2021a; Shen *et al*., 2022). Askey *et al*. (2024) have reviewed the psilocybin potential as an anti-nociceptive agent, focusing on preclinical animal models and exploring serotonergic mechanisms and neuroplastic actions that improve functional connectivity in brain regions involved in chronic pain. They also discuss its broader effects on pain and associated emotional and inflammatory components. The review by Strand *et al*. (2025) has examined psilocybin, LSD, and ketamine as potential treatments for chronic pain. It focuses on their pharmacology, effects on neuropathic pain, clinical implications, safety profiles, and patient responses.

Preclinical and clinical data also indicate that psychedelics increase the release of anti-inflammatory interleukins (e.g. IL-10) and reduce the expression and activity of other pro-inflammatory markers, including IL-6, IL-1β, tumour necrosis factor-alpha (**TNF-α**), and nuclear factor kappa B (**NF-kB**). Thus, administration of these substances may attenuate the activation of genes and downstream signalling pathways that contribute to inflammation (dos Santos, 2014; Boxler *et al*., 2018; Flanagan & Nichols, 2022; Mason *et al*., 2023; Low *et al*., 2025). DOI can inhibit TNF-α-induced inflammation by mitigating the expression of genes encoding intracellular adhesion molecule-1, vascular cell adhesion molecule-1, and IL-6 through serotonin 5-HT_2A_ receptor activation in both *in vitro* and *in vivo* (Yu *et al*., 2008; Nau *et al*., 2013). Furthermore, DOI administration blocked the activation and translocation of NF-kB and decreased nitric oxide synthase activity (Yu *et al*., 2008).

*In vitro* studies have demonstrated that psilocybin-containing mushroom extracts inhibited lipopolysaccharide (**LPS**)-induced increases in TNF-α and IL-1β, besides decreasing COX-2 concentrations in treated human U937 macrophage cells (Nkadimeng *et al*., 2021; Laabi *et al*., 2024). In healthy volunteers, a single dose of psilocybin reduced plasma levels of TNF-α immediately after administration, and IL-6 and C-reactive protein were reduced in the psilocybin group seven days later. The persisting reductions in pro-inflammatory markers correlated with clinical improvement of mood and sociability (Mason *et al*., 2023).

Possible opposite effects have been reported regarding MDMA. Acute administration of MDMA appears to promote an anti-inflammatory effect. It impairs the secretion of IL-1β and TNF-α induced by LPS administration in rodents (Connor *et al*., 2000), besides suppressing innate IFN-γ production by increasing IL-10 levels (Boyle & Connor, 2007). On the other hand, in human plasma samples collected at different time points after a single oral administration of MDMA, an increase in cortisol and lipidic mediators of inflammation was observed, suggesting stimulation of inflammatory pathways (Boxler *et al*., 2018).

Immunomodulatory effects of psychedelic substances and other chemical compounds derived from ayahuasca have also been reported (dos Santos, 2014; Galvão-Coelho *et al*., 2020). Harmine has been proposed to exert anti-inflammatory and antioxidant effects through several mechanisms, such as AMPK/Nrf2 pathway activation, reduced caspase-3 expression by repressing the Bax/Bcl2 ratio, inhibition of the c-Jun N-terminal kinase (**JNK**), downregulation of LC3B II/I, p38 MAPK, TLR4, and NF-κB levels. Furthermore, it appears to increase the expression of p62, Bcl-2, Beclin1, ULK1, and p-mTOR (Hamsa & Kuttan, 2010; Liu *et al*., 2017a; Niu *et al*., 2019; Ma *et al*., 2024; Tabaa *et al*., 2024). Harmine also attenuated bone destruction induced by an inflammatory response. It shifted the polarisation of macrophages from M1 to M2 phenotypes both *in vitro* and *in vivo* in a murine model (Wang *et al*., 2021b). A three-day ayahuasca treatment prevented anxiety and oxidative stress induced by an inflammatory insult in rats. Additionally, it increased cortical levels of the anti-inflammatory cytokine IL-4 and BDNF (de Camargo *et al*., 2024).

Although the precise molecular mechanisms related to the effects of psychedelics on immunity and inflammatory responses remain to be elucidated, the involvement of 5-HT_2A_ receptor activation has been proposed. 5-HT_2A_ receptor is widely distributed in tissues and cells, regulating innate and adaptive immune responses, such as the spleen, thymus, circulating lymphocytes, T cells, eosinophils, and mononuclear cells (Herr *et al*., 2017; Thompson & Szabo, 2020). While the 5-HT_2A_ receptor activation by 5-HT primarily contributes to inflammation, psychedelics appear to recruit anti-inflammatory intracellular signalling pathways through activation of the same receptor, possibly by stabilising it in a slightly different structural and functional conformation, that is, biased agonism (Raote *et al*., 2007; Shan *et al*., 2012; Flanagan *et al*., 2024). The anti-inflammatory effects of psychedelics resulting from the activation of the 5-HT_2A_ receptor have also been associated with improved respiratory and neurological function, demonstrating benefits in animal models of asthma (Stankevicius *et al*., 2012; Nau *et al*., 2015; Flanagan *et al*., 2019b; Flanagan *et al*., 2020) and attenuating the functional consequences of neuroinflammation (Zhong *et al*., 2015; Liu *et al*., 2017b; Sun *et al*., 2019; Nardai *et al*., 2020; Xin *et al*., 2021; Goulart da Silva *et al*., 2022; Zanikov *et al*., 2023; Zheng *et al*., 2023; Floris *et al*., 2024).

**Figure 1.**
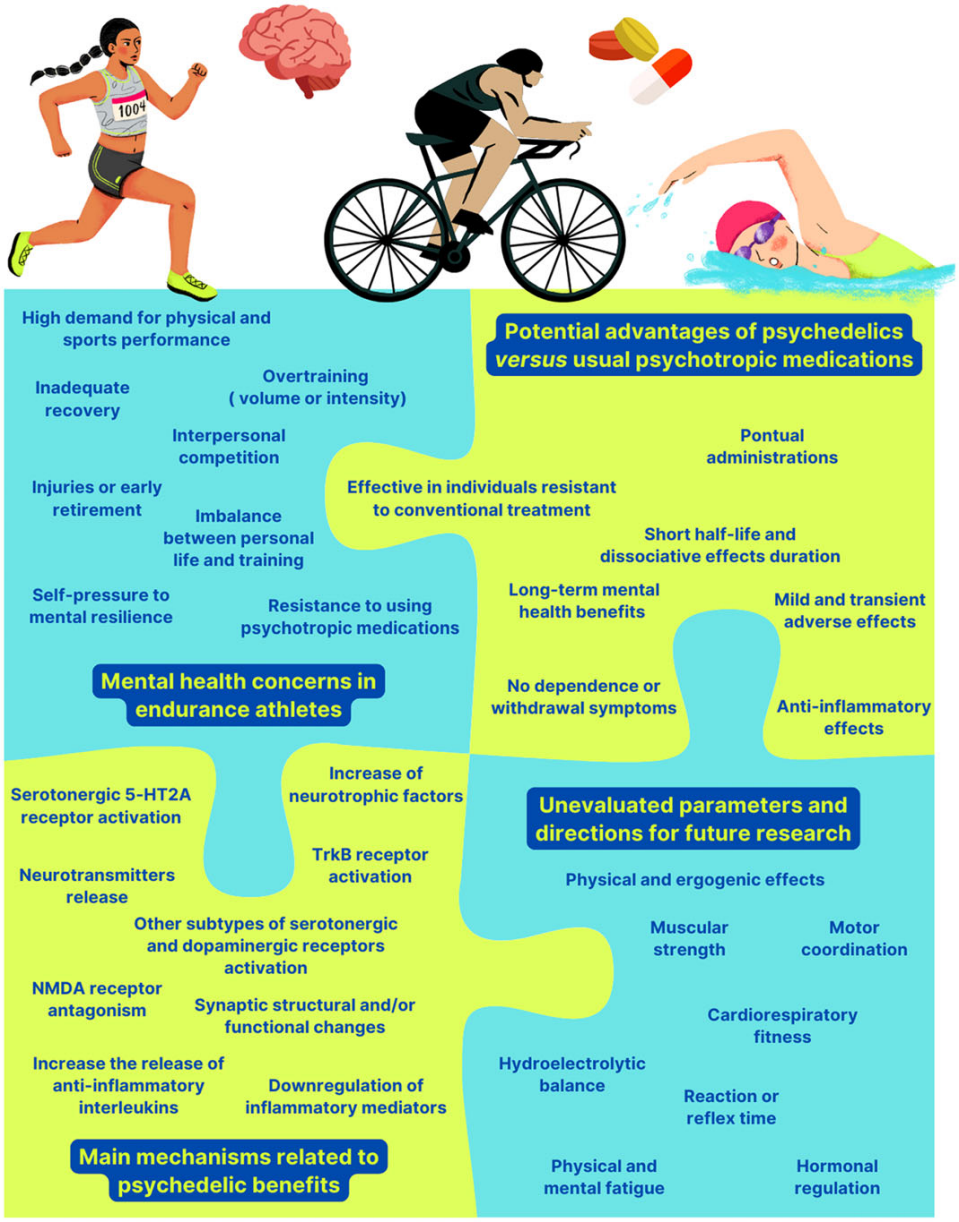
An overview of the current landscape of psychedelics and athletic performance.

A significant knowledge gap in psychedelic research, particularly regarding their potential use in athletes, is the lack of studies evaluating their effects on physical health and metabolic parameters. Based on the evidence outlined above and its potential translational implications, treating athletes with psychedelic substances may offer benefits. The improved mental well-being and emotional control associated with psychedelic therapy could contribute to performance by making them more focused and resilient. At the same time, these substances’ anti-inflammatory and analgesic effects could mitigate physical stress, reduce muscle fatigue, and facilitate recovery after prolonged or intense exercise. By reducing inflammation, psychedelics could also improve mental health and reduce symptoms in individuals with psychiatric disorders such as depression or anxiety, as convergent evidence points to an increase in inflammatory markers in these clinical conditions and the significant role of inflammation in their pathophysiology (Bauer & Teixeira, 2019; Beurel *et al*., 2020; Zeng *et al*., 2024). Since research on the use of psychedelic substances in sports contexts is incipient (Fig. 1), far more studies are needed before potentially establishing guidelines on their safe and effective use.

## Psychedelics in sports competitions: legal and regulatory considerations

Psychedelic substances have been classified as prohibited or controlled substances in most countries, posing challenges for establishing potential guidelines that ensure treatment efficacy and safety under appropriate regulatory oversight. In sports competitions, the World Anti-Doping Agency (**WADA**; https://www.wada-ama.org/en/prohibited-list) does not list psychedelics as ‘prohibited substances’, except for MDMA, which is classified as a stimulant amphetamine.

For a substance or method to be included in WADA’s Prohibited Substances List under the World Anti-Doping Code, it must meet at least two of the following three criteria: (1) it enhances or has the potential to enhance sports performance, (2) it poses an actual or potential risk to athlete health, and (3) it violates the spirit of sport as defined in the Code (https://www.wada-ama.org/en/resources/world-anti-doping-code-and-international-standards/world-anti-doping-code). To date, no clinical evidence has suggested that psychedelics act as ergogenic aids. WADA regularly updates its prohibited and restricted substances list based on evolving scientific evidence. For example, while cannabis/Δ^9^-tetrahydrocannabinol remains prohibited in competition due to its potential to impair performance, pose safety risks, and violate the ’spirit of sport’, cannabidiol (**CBD**) has been permitted, as it lacks these properties. As research on psychedelics progresses, the regulatory status of specific compounds in sports may be reevaluated, potentially leading to updates similar to the removal of CBD from the prohibited list.

## Conclusions and suggestions for future research

Several clinical studies have highlighted the mental health benefits of psychedelics and their potential role as therapeutic adjuncts to improve the quality of life, but significant considerations remain. A critical knowledge gap in evaluating these substances’ effects on physical health in humans persists. Similarly, the impact of psychedelics on physiological responses relevant to athletic performance, such as muscular strength, motor coordination, locomotion, endurance, cardiorespiratory capacity, fluid and electrolyte balance, hormonal regulation, fatigue, and reflexes, remains largely unexplored scientifically. Moreover, it is worth noting the ethical and legal concerns associated with performance-enhancing substances and the importance of distinguishing between the use of psychedelics within and outside the acute performance/sports context.

**Figure 2.**
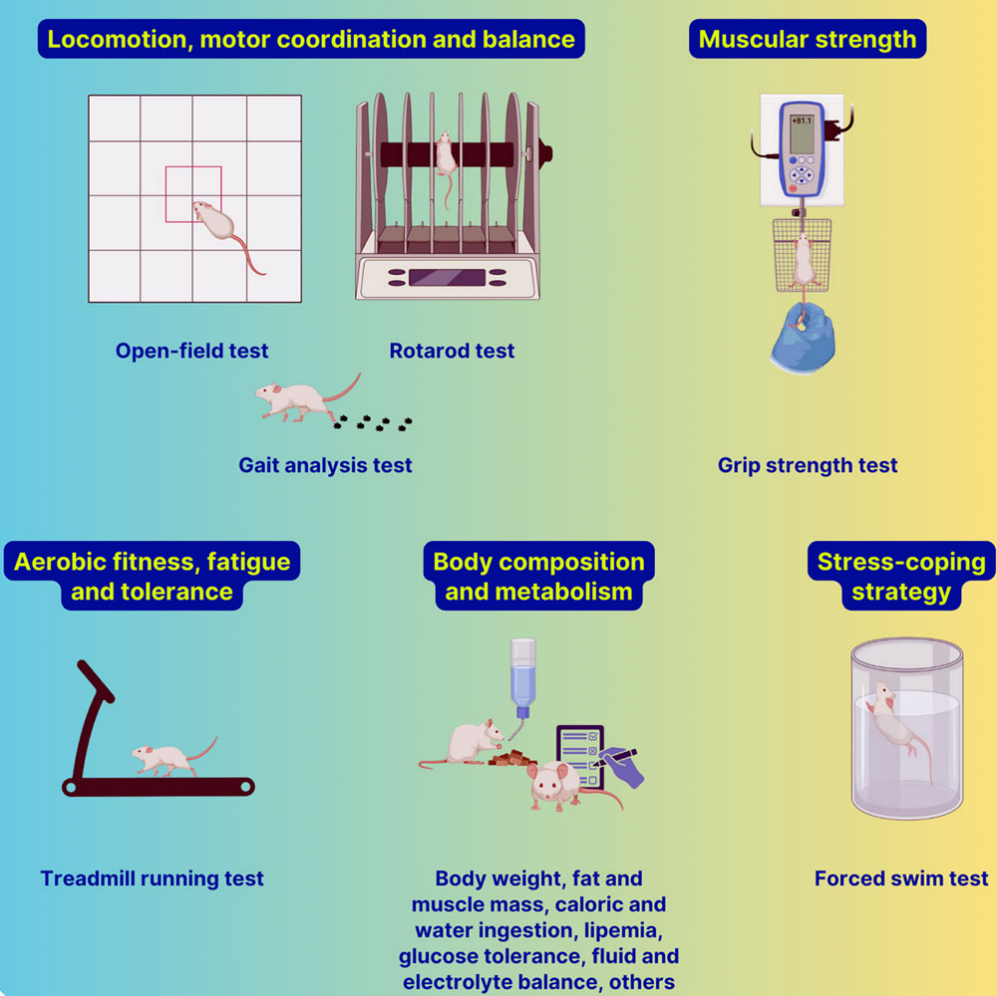
Helpful behavioural and physiological responses in rodents for inferring the physical effects of psychedelic drugs in humans.

Rodent research can provide a valuable foundation for understanding the potential effects of psychedelic therapy on physical performance in humans (Fig. 2). The rotarod test has been used to assess motor coordination and balance in rodents. The gait analysis test provides a detailed assessment of movement patterns and gait symmetry, which is crucial for identifying motor coordination changes (Carter *et al*., 2001; Deacon, 2013). Muscular strength is typically evaluated through the grip strength test, which measures the animal’s grip force by stimulating traction of the forelimbs or hind limbs (Munier *et al*., 2022). It provides a direct measure of muscle strength, relevant for assessing whether psychedelics could influence aspects of muscular endurance in humans, an essential factor in the performance of athletes. The treadmill running test (Dougherty *et al*., 2016; Castro & Kuang, 2017) is a tool for exploring the effects of psychedelic substances on endurance and cardiorespiratory capacity. Rodents are encouraged to run on a treadmill, allowing for analysis of aerobic capacity, fatigue, and prolonged exercise tolerance. These data help understand the potential of psychedelics to enhance aerobic performance and to observe possible indirect cardiorespiratory impacts from their administration. Stress resilience is also essential for high-performance athletes, and the forced swim test is a tool for assessing stress-coping strategy in rodents (Slattery & Cryan, 2012; Commons *et al*., 2017). In this test, the duration of immobility in a forced swim scenario reflects the animal’s ability to persist under adverse conditions. Several psychedelics have been shown to decrease immobility and increase active behaviours, including swimming and climbing (Cameron *et al*., 2018; Hibicke *et al*., 2020; Odland *et al*., 2022; Rakoczy *et al*., 2024). Metabolic parameters and overall physical condition can be monitored through assessments such as food and water intake (to evaluate impacts on basal metabolism and caloric needs) and body condition scoring, which provides a qualitative assessment of the animal’s overall physical state by monitoring body composition and body mass index.

Overall, these methods could provide preclinical evidence to elucidate the influence of psychedelics on motor, metabolic, and cardiorespiratory functions, as well as their impact on stress resilience. This knowledge could inform the design of safer and more effective clinical protocols to explore the potential benefits of psychedelics as adjunctive therapies in enhancing the mental health and physical performance of athletes and non-athletes. Such studies could also illuminate the underlying mechanisms of action, identify potential effects on organs and tissues beyond the central nervous system, and investigate potential sex differences or genetic and metabolic influences (Rakoczy *et al*., 2024; Werle & Bertoglio, 2024).

## Footnotes

1 It is a psychoactive brew from the Amazon, typically made from *Banisteriopsis caapi* and *Psychotria viridis*. The β-carbolines harmine, harmaline, and tetrahydroharmine present in *B*. *caapi* act as monoamine oxidase inhibitors, preventing the first-pass metabolism of DMT found in *P*. *viridis* and allowing it to become orally active. It is also worth noting that DMT is found in various other plants worldwide.

## References

[ref1] Aaronson ST , van der Vaart A , Miller T , LaPratt J , Swartz K , Shoultz A , Lauterbach M , Sackeim HA and Suppes T (2024) Single-dose synthetic psilocybin with psychotherapy for treatment-resistant bipolar type II major depressive episodes: a nonrandomized open-label trial. JAMA Psychiatry 81(6), 555–562. DOI: 10.1001/jamapsychiatry.2023.4685. 38055270 PMC10701666

[ref2] Allen N , Jeremiah A , Murphy R , Sumner R , Forsyth A , Hoeh N , Menkes DB , Evans W , Muthukumaraswamy S , Sundram F and Roop P (2024) LSD increases sleep duration the night after microdosing. Translational Psychiatry 14(1), 191. DOI: 10.1038/s41398-024-02900-4. 38622150 PMC11018829

[ref3] Arruda Sanchez T , Ramos LR , Araujo F , Schenberg EE , Yonamine M , Lobo I , de Araujo DB and Luna LE (2024) 2024Emotion regulation effects of ayahuasca in experienced subjects during implicit aversive stimulation: an fMRI study. Journal of Ethnopharmacology 320, 117430. DOI: 10.1016/j.jep.2023.117430. 37979818

[ref4] Askey T , Lasrado R , Maiarú M and Stephens GJ (2024) Psilocybin as a novel treatment for chronic pain. British Journal of Pharmacology. DOI: 10.1111/bph.17420. 39614355

[ref5] Atila C , Straumann I , Vizeli P , Beck J , Monnerat S , Holze F , Liechti ME and Christ-Crain M (2024) Oxytocin and the role of fluid restriction in MDMA-induced hyponatremia: a secondary analysis of 4 randomized clinical trials. JAMA Network Open 7(11), e2445278. DOI: 10.1001/jamanetworkopen.2024.45278. 39546312 PMC11568463

[ref6] Bacqué-Cazenave J , Bharatiya R , Barrière G , Delbecque JP , Bouguiyoud N , Di Giovanni G , Cattaert D and De Deurwaerdère P (2020) Serotonin in animal cognition and behavior. International Journal of Molecular Sciences 21(5), 1649. DOI: 10.3390/ijms21051649. 32121267 PMC7084567

[ref7] Barber GS and Aaronson ST (2022) The emerging field of psychedelic psychotherapy. Current Psychiatry Reports 24(10), 583–590. DOI: 10.1007/s11920-022-01363-y. 36129571 PMC9553847

[ref8] Barrett FS , Doss MK , Sepeda ND , Pekar JJ and Griffiths RR (2020) Emotions and brain function are altered up to one month after a single high dose of psilocybin. Scientific Reports 10(1), 2214. DOI: 10.1038/s41598-020-59282-y. 32042038 PMC7010702

[ref9] Bauer ME and Teixeira AL (2019) Inflammation in psychiatric disorders: what comes first? Annals of the New York Academy of Sciences 1437(1), 57–67. DOI: 10.1111/nyas.13712. 29752710

[ref10] Beable SE (2024) Depressive disorders in athletes. Clinical Journal of Sport Medicine 43(1), 53–70. DOI: 10.1016/j.csm.2023.06.011. 37949514

[ref11] Berger NJA , Best R , Best AW , Lane AM , Millet GY , Barwood M , Marcora S , Wilson P and Bearden S (2024) Limits of ultra: towards an interdisciplinary understanding of ultra-endurance running performance. Sports Medicine 54(1), 73–93. DOI: 10.1007/s40279-023-01936-8. 37751076

[ref12] Beurel E , Toups M and Nemeroff CB (2020) The bidirectional relationship of depression and inflammation: double trouble. Neuron 107(2), 234–256. DOI: 10.1016/j.neuron.2020.06.002. 32553197 PMC7381373

[ref13] Bhatt KV and Weissman CR (2024) The effect of psilocybin on empathy and prosocial behavior: a proposed mechanism for enduring antidepressant effects. Mental Health Research 3(1), 7. DOI: 10.1038/s44184-023-00053-8. PMC10955966 38609500

[ref14] Bogenschutz MP , Forcehimes AA , Pommy JA , Wilcox CE , Barbosa PC and Strassman RJ (2015) Psilocybin-assisted treatment for alcohol dependence: a proof-of-concept study. Journal of Psychopharmacology 29(3), 289–299. DOI: 10.1177/0269881114565144. 25586396

[ref15] Bogenschutz MP , Ross S , Bhatt S , Baron T , Forcehimes AA , Laska E , Mennenga SE , O’Donnell K , Owens LT , Podrebarac S , Rotrosen J , Tonigan JS and Worth L (2022) Percentage of heavy drinking days following psilocybin-assisted psychotherapy vs placebo in the treatment of adult patients with alcohol use disorder: a randomized clinical trial. JAMA Psychiatry 79(10), 953–962. DOI: 10.1001/jamapsychiatry.2022.2096. 36001306 PMC9403854

[ref16] Bomfim JHGG (2020) Pharmaceutical care in sports. Pharmacy (Basel) 8(4), 218. DOI: 10.3390/pharmacy8040218. 33207610 PMC7712766

[ref17] Boxler MI , Streun GL , Liechti ME , Schmid Y , Kraemer T and Steuer AE (2018) Human metabolome changes after a single dose of 3,4-methylenedioxymethamphetamine (MDMA) with special focus on steroid metabolism and inflammation processes. Journal of Proteome Research 17(8), 2900–2907. DOI: 10.1021/acs.jproteome.8b00438. 29947220

[ref18] Boyle NT and Connor TJ (2007) MDMA (“Ecstasy”) suppresses the innate IFN-gamma response in vivo: a critical role for the anti-inflammatory cytokine IL-10. European Journal of Pharmacology 572(2-3), 228–238. DOI: 10.1016/j.ejphar.2007.07.020. 17689526

[ref19] Breeksema JJ , Niemeijer A , Krediet E , Karsten T , Kamphuis J , Vermetten E , van den Brink W and Schoevers R (2024) Patient perspectives and experiences with psilocybin treatment for treatment-resistant depression: a qualitative study. Scientific Reports 14(1), 2929. DOI: 10.1038/s41598-024-53188-9. 38316896 PMC10844281

[ref20] Brewerton TD , Wang JB , Lafrance A , Pamplin C , Mithoefer M , Yazar-Klosinki B , Emerson A and Doblin R (2022) MDMA-assisted therapy significantly reduces eating disorder symptoms in a randomized placebo-controlled trial of adults with severe PTSD. Journal of Psychiatric Research 149, 128–135. DOI: 10.1016/j.jpsychires.2022.03.008. 35272210

[ref21] Calder AE and Hasler G (2023) Towards an understanding of psychedelic-induced neuroplasticity. Neuropsychopharmacology 48(1), 104–112. DOI: 10.1038/s41386-022-01389-z. 36123427 PMC9700802

[ref22] Cameron LP , Benetatos J , Lewis V , Bonniwell EM , Jaster AM , Moliner R , Castrén E , McCorvy JD , Palner M and Aguilar-Valles A (2023) Beyond the 5-HT_2A_ receptor: classic and nonclassic targets in psychedelic drug action. Journal of Neuroscience 43(45), 7472–7482. DOI: 10.1523/JNEUROSCI.1384-23.2023. 37940583 PMC10634557

[ref23] Cameron LP , Benson CJ , Dunlap LE and Olson DE (2018) Effects of N, N-dimethyltryptamine on rat behaviors relevant to anxiety and depression. ACS Chemical Neuroscience 9(7), 1582–1590. DOI: 10.1021/acschemneuro.8b00134. 29664276 PMC7196340

[ref24] Carhart-Harris R , Giribaldi B , Watts R , Baker-Jones M , Murphy-Beiner A , Murphy R , Martell J , Blemings A , Erritzoe D and Nutt DJ (2021) Trial of psilocybin versus escitalopram for depression. New England Journal of Medicine 384(15), 1402–1411. DOI: 10.1056/NEJMoa2032994. 33852780

[ref25] Carhart-Harris RL , Bolstridge M , Day CMJ , Rucker J , Watts R , Erritzoe DE , Kaelen M , Giribaldi B , Bloomfield M , Pilling S , Rickard JA , Forbes B , Feilding A , Taylor D , Curran HV and Nutt DJ (2018) Psilocybin with psychological support for treatment-resistant depression: six-month follow-up. Psychopharmacology 235(2), 399–408. DOI: 10.1007/s00213-017-4771-x. 29119217 PMC5813086

[ref26] Carhart-Harris RL , Bolstridge M , Rucker J , Day CM , Erritzoe D , Kaelen M , Bloomfield M , Rickard JA , Forbes B , Feilding A , Taylor D , Pilling S , Curran VH and Nutt DJ (2016) Psilocybin with psychological support for treatment-resistant depression: an open-label feasibility study. Lancet Psychiatry 3(7), 619–627. DOI: 10.1016/S2215-0366(16)30065-7. 27210031

[ref27] Carhart-Harris RL , Erritzoe D , Williams T , Stone JM , Reed LJ , Colasanti A , Tyacke RJ , Leech R , Malizia AL , Murphy K , Hobden P , Evans J , Feilding A , Wise RG and Nutt DJ (2012) Neural correlates of the psychedelic state as determined by fMRI studies with psilocybin. National Academy of Sciences U.S.A 109(6), 2138–2143. DOI: 10.1073/pnas.1119598109-10.1073/pnas.1119598109. PMC3277566 22308440

[ref28] Carhart-Harris RL and Goodwin GM (2017) The therapeutic potential of psychedelic drugs: past, present, and future. Neuropsychopharmacology 42(11), 2105–2113. DOI: 10.1038/npp.2017.84. 28443617 PMC5603818

[ref29] Carhart-Harris RL , Muthukumaraswamy S , Roseman L , Kaelen M , Droog W , Murphy K , Tagliazucchi E , Schenberg EE , Nest T , Orban C , Leech R , Williams LT , Williams TM , Bolstridge M , Sessa B , McGonigle J , Sereno MI , Nichols D , Hellyer PJ , Hobden P , Evans J , Singh KD , Wise RG , Curran HV , Feilding A and Nutt DJ (2016) Neural correlates of the LSD experience revealed by multimodal neuroimaging. National Academy of Sciences U.S.A 113(17), 4853–4858. DOI: 10.1073/pnas.1518377113. PMC4855588 27071089

[ref30] Carhart-Harris RL , Roseman L , Bolstridge M , Demetriou L , Pannekoek JN , Wall MB , Tanner M , Kaelen M , McGonigle J , Murphy K , Leech R , Curran HV and Nutt DJ (2017) Psilocybin for treatment-resistant depression: fMRI-measured brain mechanisms. Scientific Reports 7(1), 13187. DOI: 10.1038/s41598-017-13282-7. 29030624 PMC5640601

[ref31] Carter RJ , Morton J and Dunnett SB (2001) Motor coordination and balance in rodents. Current Protocols in Neuroscience 8(8), 12. DOI: 10.1002/0471142301.ns0812s15. 18428540

[ref32] Castro B and Kuang S (2017) Evaluation of muscle performance in mice by treadmill exhaustion test and whole-limb grip strength assay. Bio Protocol 7(8), e2237. DOI: 10.21769/BioProtoc.2237. PMC5510664 28713848

[ref33] Cavarra M , Falzone A , Ramaekers JG , Kuypers KPC and Mento C (2022) Psychedelic-assisted psychotherapy-A systematic review of associated psychological interventions. Frontiers of Psychology 13, 887255. DOI: 10.3389/fpsyg.2022.887255. PMC9226617 35756295

[ref34] Chang C , Putukian M , Aerni G , Diamond A , Hong G , Ingram Y , Reardon CL and Wolanin A (2020) Mental health issues and psychological factors in athletes: detection, management, effect on performance and prevention: American medical society for sports medicine position statement-executive summary. British Journal of Sports Medicine 54(4), 216–220. DOI: 10.1136/bjsports-2019-101583. 31810972

[ref35] Ching TH , Williams MT , Wang JB , Jerome L , Yazar-Klosinski B , Emerson A and Doblin R (2022) MDMA-assisted therapy for posttraumatic stress disorder: a pooled analysis of ethnoracial differences in efficacy and safety from two phase 2 open-label lead-in trials and a phase 3 randomized, blinded placebo-controlled trial. Journal of Psychopharmacology 36(8), 974–986. DOI: 10.1177/02698811221104052. 35727042

[ref36] Commons KG , Cholanians AB , Babb JA and Ehlinger DG (2017) The rodent forced swim test measures stress-coping strategy, not depression-like behavior. ACS Chemical Neuroscience 8(5), 955–960. DOI: 10.1021/acschemneuro.7b00042. 28287253 PMC5518600

[ref37] Connor TJ , Kelly JP , McGee M and Leonard BE (2000) Methylenedioxymethamphetamine (MDMA; ecstasy) suppresses IL-1beta and TNF-alpha secretion following an in vivo lipopolysaccharide challenge. Life Science 67(13), 1601–1612. DOI: 10.1016/s0024-3205(00)00743-8. 10983854

[ref38] Cuerva K , Spirou D , Cuerva A , Delaquis C and Raman J (2024) Perspectives and preliminary experiences of psychedelics for the treatment of eating disorders: a systematic scoping review. European Eating Disorders Review 32(5), 980–1001. DOI: 10.1002/erv.3101. 38783636

[ref56] D’Souza DC , Syed SA , Flynn LT , Safi-Aghdam H , Cozzi NV and Ranganathan M (2022) Exploratory study of the dose-related safety, tolerability, and efficacy of dimethyltryptamine (DMT) in healthy volunteers and major depressive disorder. Neuropsychopharmacology 47(10), 1854–1862. DOI: 10.1038/s41386-022-01344-y. 35660802 PMC9372173

[ref39] Danforth AL , Grob CS , Struble C , Feduccia AA , Walker N , Jerome L , Yazar-Klosinski B and Emerson A (2018) Reduction in social anxiety after MDMA-assisted psychotherapy with autistic adults: a randomized, double-blind, placebo-controlled pilot study. Psychopharmacology (Berl) 235(11), 3137–3148. DOI: 10.1007/s00213-018-5010-9. 30196397 PMC6208958

[ref40] Darke S , Duflou J , Peacock A , Farrell M , Hall W and Lappin J (2024) A retrospective study of the characteristics and toxicology of cases of lysergic acid diethylamide (LSD)- and psilocybin-related death in Australia. Addiction 119(9), 1564–1571. DOI: 10.1111/add.16518. 38771189

[ref41] Davis AK , Barrett FS , May DG , Cosimano MP , Sepeda ND , Johnson MW , Finan PH and Griffiths RR (2021) Effects of psilocybin-assisted therapy on major depressive disorder: a randomized clinical trial. JAMA Psychiatry 78(5), 481–489. DOI: 10.1001/jamapsychiatry.2020.3285. 33146667 PMC7643046

[ref42] Daws RE , Timmermann C , Giribaldi B , Sexton JD , Wall MB , Erritzoe D , Roseman L , Nutt D and Carhart-Harris R (2022) Increased global integration in the brain after psilocybin therapy for depression. Nature Medicine 28(4), 844–851. DOI: 10.1038/s41591-022-01744-z. 35411074

[ref43] de Almeida RN , Galvão ACM , da Silva FS , Silva EADS , Palhano-Fontes F , Maia-de-Oliveira JP , de Araújo LB , Lobão-Soares B and Galvão-Coelho NL (2019) Modulation of serum brain-derived neurotrophic factor by a single dose of ayahuasca: observation from a randomized controlled trial. Frontiers of Psychology 10, 1234. DOI: 10.3389/fpsyg.2019.01234. PMC6558429 31231276

[ref44] de Bragança AC , Moreau RLM , de Brito T , Shimizu MHM , Canale D , de Jesus DA , Silva AMG , Gois PH , Seguro AC and Magaldi AJ (2017) Ecstasy induces reactive oxygen species, kidney water absorption and rhabdomyolysis in normal rats. Effect of N-acetylcysteine and allopurinol in oxidative stress and muscle fiber damage. PLoS One 12(7), e0179199. DOI: 10.1371/journal.pone.0179199. 28678861 PMC5497951

[ref45] de Camargo RW , Joaquim L , Machado RS , de Souza Ramos S , da Rosa LR , de Novais Junior LR , Mathias K , Maximiano L , Strickert YR , Nord R , Gava ML , Scarpari E , Martins HM , Lins EMF , Chaves JS , da Silva LE , de Oliveira MP , da Silva MR , Fernandes BB , Tiscoski ADB , Piacentini N , Santos FP , Inserra A , Bobinski F , Rezin GT , Yonamine M , Petronilho F and de Bitencourt RM (2024, Ayahuasca pretreatment prevents sepsis-induced anxiety-like behavior, neuroinflammation, and oxidative stress, and increases brain-derived neurotrophic factor. Molecular neurobiology, Ayahuasca Pretreatment Prevents Sepsis-Induced Anxiety-Like Behavior, Neuroinflammation, and Oxidative Stress, and Increases Brain-Derived Neurotrophic Factor. Molecular Neurobiology. DOI: 10.1007/s12035-024-04597-4. 39613951

[ref46] De Gregorio D , Popic J , Enns JP , Inserra A , Skalecka A , Markopoulos A , Posa L , Lopez-Canul M , He Q , Lafferty CK , Britt JP , Comai S , Aguilar-Valles A , Sonenberg N and Gobbi G (2021) Lysergic acid diethylamide (LSD) promotes social behavior through mTORC1 in the excitatory neurotransmission. Proceedings of the National Academy of Sciences of the United States of America 118(5), e20207051188. DOI: 10.1073/pnas.2020705118. PMC7865169 33495318

[ref47] de Vos CMH , Mason NL and Kuypers KPC (2021) Psychedelics and neuroplasticity: a systematic review unraveling the biological underpinnings of psychedelics. Frontiers of Psychology 12, 724606. DOI: 10.3389/fpsyt.2021.724606. PMC8461007 34566723

[ref48] Deacon RM (2013) Measuring motor coordination in mice. Journal of Visualized Experiments 75(75), e2609. DOI: 10.3791/2609. PMC3724562 23748408

[ref52] dos Santos RG (2014) Immunological effects of ayahuasca in humans. Journal of Psychoactive Drugs 46(5), 383–388. DOI: 10.1080/02791072.2014.960113. 25364989

[ref50] Dos Santos RG and Hallak JEC (2024) Ayahuasca: pharmacology, safety, and therapeutic effects. CNS Spectrums 20(1), 1–9. DOI: 10.1017/s109285292400213x. PMC13064705 39564645

[ref51] Dos Santos RG , Osório FL , Rocha JM , Rossi GN , Bouso JC , Rodrigues LS , de Oliveira Silveira G , Yonamine M and Hallak JEC (2021) Ayahuasca improves self-perception of speech performance in subjects with social anxiety disorder: a pilot, proof-of-concept, randomized, Placebo-Controlled Trial. Journal of Clinical Psychopharmacology 41(5), 540–550. DOI: 10.1097/JCP.0000000000001428. 34166299

[ref53] Doss MK , DeMarco A , Dunsmoor JE , Cisler JM , Fonzo GA and Nemeroff CB (2024) How psychedelics modulate multiple memory mechanisms in posttraumatic stress disorder. Drugs 84(11), 1419–1443. DOI: 10.1007/s40265-024-02106-4. 39455547

[ref54] Dougherty JP , Springer DA and Gershengorn MC (2016) The treadmill fatigue test: a simple, high-throughput assay of fatigue-like behavior for the mouse. Journal of Visualized Experiments 31(111), 54052. DOI: 10.3791/54052. PMC4927751 27286034

[ref55] Doyle AJ , Meyer J , Breen K and Hunt BJ (2020) N-methyl-3,4-methylendioxymethamphetamine (MDMA)-related coagulopathy and rhabdomyolysis: a case series and literature review. Research and Practice in Thrombosis and Haemostasis 4(5), 829–834. DOI: 10.1002/rth2.12360. 32685891 PMC7354411

[ref57] Duarte JA , Leão A , Magalhães J , Ascensão A , Bastos ML , Amado FL , Vilarinho L , Quelhas D , Appell HJ and Carvalho F (2005) Strenuous exercise aggravates MDMA-induced skeletal muscle damage in mice. Toxicology 206(3), 349–358. DOI: 10.1016/j.tox.2004.07.012. 15588925

[ref59] Dumont GJ , Sweep FC , van der Steen R , Hermsen R , Donders AR , Touw DJ , van Gerven JM , Buitelaar JK and Verkes RJ (2009) Increased oxytocin concentrations and prosocial feelings in humans after ecstasy (3,4-methylenedioxymethamphetamine) administration. Society for Neuroscience 4(4), 359–366. DOI: 10.1080/17470910802649470. 19562632

[ref60] Edwards CD (2024) Management of mental health challenges in athletes: screening, pharmacology, and behavioral approaches. Clinical Sports Medicine 43(1), 13–31. DOI: 10.1016/j.csm.2023.06.006. 37949507

[ref61] Fadahunsi N , Lund J , Breum AW , Mathiesen CV , Larsen IB , Knudsen GM , Klein AB and Clemmensen C (2022) Acute and long-term effects of psilocybin on energy balance and feeding behavior in mice. Translational Psychiatry 12(1), 330. DOI: 10.1038/s41398-022-02103-9. 35953488 PMC9372155

[ref62] Family N , Hendricks PS , Williams LT , Luke D , Krediet E , Maillet EL and Raz S (2022) Safety, tolerability, pharmacokinetics, and subjective effects of 50, 75, and 100 μg LSD in healthy participants within a novel intervention paradigm: a proof-of-concept study. Journal of Psychopharmacology 36(3), 321–336. DOI: 10.1177/02698811211069103. 35253516

[ref63] Flaive A , Fougère M , van der Zouwen CI and Ryczko D (2020) Serotonergic modulation of locomotor activity from basal vertebrates to mammals. Frontiers Neural Circuits 14, 590299. DOI: 10.3389/fncir.2020.590299. PMC7674590 33224027

[ref64] Flanagan TW , Billac GB , Landry AN , Sebastian MN , Cormier SA and Nichols CD (2020) Structure-activity relationship analysis of psychedelics in a rat model of asthma reveals the anti-inflammatory pharmacophore. ACS Pharmacology & Translational Science 4(2), 488–502. DOI: 10.1021/acsptsci.0c00063. 33860179 PMC8033619

[ref65] Flanagan TW , Foster TP , Galbato TE , Lum PY , Louie B , Song G , Halberstadt AL , Billac GB and Nichols CD (2024) Serotonin-2 receptor agonists produce anti-inflammatory effects through functionally selective mechanisms that involve the suppression of disease-induced Arginase 1 expression. ACS Pharmacology & Translational Science 7(2), 478–492. DOI: 10.1021/acsptsci.3c00297. 38357283 PMC10863441

[ref66] Flanagan TW and Nichols CD (2022) Psychedelics and anti-inflammatory activity in animal models. Current Topics in Behavioral Neurosciences 56, 229–245. DOI: 10.1007/7854_2022_367. 35546383

[ref68] Flanagan TW , Sebastian MN , Battaglia DM , Foster TP , Maillet EL and Nichols CD (2019a) Activation of 5-HT_2_ receptors reduces inflammation in vascular tissue and cholesterol levels in high-fat diet-fed apolipoprotein E knockout mice. Scientific Reports 9(1), 13444. DOI: 10.1038/s41598-019-49987-0. 31530895 PMC6748996

[ref67] Flanagan TW , Sebastian MN , Battaglia DM , Foster TP , Cormier SA and Nichols CD (2019b) 5-HT_2_ receptor activation alleviates airway inflammation and structural remodeling in a chronic mouse asthma model. Life Science 236, 116790. DOI: 10.1016/j.lfs.2019.116790. 31626791

[ref69] Floris G , Dabrowski KR , Zanda MT and Daws SE (2024) Psilocybin reduces heroin seeking behavior and modulates inflammatory gene expression in the nucleus accumbens and prefrontal cortex of male rats. Molecular Psychiatry 10.1038/s41380-024-02788-y. PMC12015112 39433903

[ref70] Freidel N , Kreuder L , Rabinovitch BS , Chen FY , Huang RST and Lewis EC (2024) Psychedelics, epilepsy, and seizures: a review. Frontiers of Psychology 14, 1326815. DOI: 10.3389/fphar.2023.1326815. PMC10811552 38283836

[ref71] Galvão ACM , de Almeida RN , Silva EADS , Freire FAM , Palhano-Fontes F , Onias H , Arcoverde E , Maia-de-Oliveira JP , de Araújo DB , Lobão-Soares B and Galvão-Coelho NL (2018) Cortisol modulation by Ayahuasca in patients with treatment resistant depression and healthy controls. Frontiers of Psychology 9, 185. DOI: 10.3389/fpsyt.2018.00185. PMC5952178 29867608

[ref72] Galvão-Coelho NL , de Menezes Galvão AC , de Almeida RN , Palhano-Fontes F , Campos Braga I , Lobão Soares B , Maia-de-Oliveira JP , Perkins D , Sarris J and de Araujo DB (2020) Changes in inflammatory biomarkers are related to the antidepressant effects of Ayahuasca. Journal of Psychopharmacology 34(10), 1125–1133. DOI: 10.1177/0269881120936486. 32648790

[ref73] Gasser P , Holstein D , Michel Y , Doblin R , Yazar-Klosinski B , Passie T and Brenneisen R (2014) Safety and efficacy of lysergic acid diethylamide-assisted psychotherapy for anxiety associated with life-threatening diseases. Journal of Nervous and Mental Disease 202(7), 513–520. DOI: 10.1097/NMD.0000000000000113. 24594678 PMC4086777

[ref74] Gasser P , Kirchner K and Passie T (2015) LSD-assisted psychotherapy for anxiety associated with a life-threatening disease: a qualitative study of acute and sustained subjective effects. Journal of Psychopharmacology 29(1), 57–68. DOI: 10.1177/0269881114555249. 25389218

[ref75] Glick ID , Stillman MA and McDuff D (2020) Update on integrative treatment of psychiatric symptoms and disorders in athletes. Physician and Sportsmedicine 48(4), 385–391. DOI: 10.1080/00913847.2020.1757370. 32298189

[ref76] Glick ID , Stillman MA , Reardon CL and Ritvo EC (2012) Managing psychiatric issues in elite athletes. Journal of Clinical Psychiatry 73(5), 640–644. DOI: 10.4088/jcp.11r07381. 22697190

[ref77] Golchoobian R , Nabavizadeh F , Roghani M , Foroumadi A and Mohammadian M (2017) Alleviates MDMA-induced disturbance of serum glucose and lipids levels in the rat. Acta Medica Iranica 55(12), 736–743. 29373879

[ref78] Goodwin GM , Aaronson ST , Alvarez O , Arden PC , Baker A , Bennett JC , Bird C , Blom RE , Brennan C , Brusch D , Burke L , Campbell-Coker K , Carhart-Harris R , Cattell J , Daniel A , DeBattista C , Dunlop BW , Eisen K , Feifel D , Forbes M , Haumann HM , Hellerstein DJ , Hoppe AI , Husain MI , Jelen LA , Kamphuis J , Kawasaki J , Kelly JR , Key RE , Kishon R , Knatz Peck S , Knight G , Koolen MHB , Lean M , Licht RW , Maples-Keller JL , Mars J , Marwood L , McElhiney MC , Miller TL , Mirow A , Mistry S , Mletzko-Crowe T , Modlin LN , Nielsen RE , Nielson EM , Offerhaus SR , O’Keane V , Páleníček T , Printz D , Rademaker MC , van Reemst A , Reinholdt F , Repantis D , Rucker J , Rudow S , Ruffell S , Rush AJ , Schoevers RA , Seynaeve M , Shao S , Soares JC , Somers M , Stansfield SC , Sterling D , Strockis A , Tsai J , Visser L , Wahba M , Williams S , Young AH , Ywema P , Zisook S and Malievskaia E (2022) Single-dose psilocybin for a treatment-resistant episode of major depression. New England Journal of Medicine 387(18), 1637–1648. DOI: 10.1056/NEJMoa2206443. 36322843

[ref79] Goodwin GM , Croal M , Feifel D , Kelly JR , Marwood L , Mistry S , O’Keane V , Peck SK , Simmons H , Sisa C , Stansfield SC , Tsai J , Williams S and Malievskaia E (2023) Psilocybin for treatment resistant depression in patients taking a concomitant SSRI medication. Neuropsychopharmacology 48(10), 1492–1499. DOI: 10.1038/s41386-023-01648-7. 37443386 PMC10425429

[ref80] Gorman I , Belser AB , Jerome L , Hennigan C , Shechet B , Hamilton S , Yazar-Klosinski B , Emerson A and Feduccia AA (2020) Posttraumatic growth after MDMA-assisted psychotherapy for posttraumatic stress disorder. Journal of Traumatic Stress 33(2), 161–170. DOI: 10.1002/jts.22479. 32073177 PMC7216948

[ref81] Goulart da Silva M , Daros GC , Santos FP , Yonamine M and de Bitencourt RM (2022) Antidepressant and anxiolytic-like effects of ayahuasca in rats subjected to LPS-induced neuroinflammation. Behavioural Brain Research 434, 114007. DOI: 10.1016/j.bbr.2022.114007. 35843462

[ref82] Gouttebarge V , Castaldelli-Maia JM , Gorczynski P , Hainline B , Hitchcock ME , Kerkhoffs GM , Rice SM and Reardon CL (2019) Occurrence of mental health symptoms and disorders in current and former elite athletes: a systematic review and meta-analysis. British Journal of Sports Medicine 53(11), 700–706. DOI: 10.1136/bjsports-2019-100671. 31097451 PMC6579497

[ref83] Graham DL , Herring NR , Schaefer TL , Vorhees CV and Williams MT (2010) Glucose and corticosterone changes in developing and adult rats following exposure to (+/-)-3,4-methylendioxymethamphetamine or 5-methoxydiisopropyltryptamine. Neurotoxicology and Teratology 32(2), 152–157. DOI: 10.1016/j.ntt.2009.08.012. 19737610 PMC2839063

[ref84] Griffiths RR , Johnson MW , Carducci MA , Umbricht A , Richards WA , Richards BD , Cosimano MP and Klinedinst MA (2016) Psilocybin produces substantial and sustained decreases in depression and anxiety in patients with life-threatening cancer: a randomized double-blind trial. Journal of Psychopharmacology 30(12), 1181–1197. DOI: 10.1177/0269881116675513. 27909165 PMC5367557

[ref85] Griffiths RR , Johnson MW , Richards WA , Richards BD , Jesse R , MacLean KA , Barrett FS , Cosimano MP and Klinedinst MA (2018) Psilocybin-occasioned mystical-type experience in combination with meditation and other spiritual practices produces enduring positive changes in psychological functioning and in trait measures of prosocial attitudes and behaviors. Journal of Psychopharmacology 32(1), 49–69. DOI: 10.1177/0269881117731279. 29020861 PMC5772431

[ref86] Griffiths RR , Johnson MW , Richards WA , Richards BD , McCann U and Jesse R (2011) Psilocybin occasioned mystical-type experiences: immediate and persisting dose-related effects. Psychopharmacology (Berl) 218(4), 649–665. DOI: 10.1007/s00213-011-2358-5. 21674151 PMC3308357

[ref87] Grob CS , Danforth AL , Chopra GS , Hagerty M , McKay CR , Halberstadt AL and Greer GR (2011) Pilot study of psilocybin treatment for anxiety in patients with advanced-stage cancer. Archives Of General Psychiatry 68(1), 71–78. DOI: 10.1001/archgenpsychiatry.2010.116. 20819978

[ref88] Gukasyan N , Davis AK , Barrett FS , Cosimano MP , Sepeda ND , Johnson MW and Griffiths RR (2022) Efficacy and safety of psilocybin-assisted treatment for major depressive disorder: prospective 12-month follow-up. Journal of Psychopharmacology 36(2), 151–158. DOI: 10.1177/02698811211073759. 35166158 PMC8864328

[ref89] Haden M and Woods B (2020) LSD overdoses: three case reports. Journal of Studies on Alcohol and Drugs 81(1), 115–118. DOI: 10.15288/jsad.2020.81.115. 32048609

[ref90] Halachanova V , Sansone RA and McDonald S (2001) Delayed rhabdomyolysis after ecstasy use. Mayo Clinic Proceedings 76(1), 112–113. DOI: 10.4065/76.1.112. 11155406

[ref91] Halman A , Kong G , Sarris J and Perkins D (2024) Drug-drug interactions involving classic psychedelics: a systematic review. Journal of Psychopharmacology 38(1), 3–18. DOI: 10.1177/02698811231211219. 37982394 PMC10851641

[ref92] Hamsa TP and Kuttan G (2010) Harmine inhibits tumour specific neo-vessel formation by regulating VEGF, MMP, TIMP and pro-inflammatory mediators both in vivo and in vitro. European Journal of Pharmacology 649(1-3), 64–73. DOI: 10.1016/j.ejphar.2010.09.010. 20858484

[ref93] He DY , McGough NN , Ravindranathan A , Jeanblanc J , Logrip ML , Phamluong K , Janak PH and Ron D (2005) Glial cell line-derived neurotrophic factor mediates the desirable actions of the anti-addiction drug ibogaine against alcohol consumption. Journal of Neurosciences 25(3), 619–628. DOI: 10.1523/JNEUROSCI.3959-04.2005. PMC1193648 15659598

[ref94] Heresco-Levy U and Lerer B (2024) Synergistic psychedelic - NMDAR modulator treatment for neuropsychiatric disorders. Molecular Psychiatry 29(1), 146–152. DOI: 10.1038/s41380-023-02312-8. 37945694

[ref95] Herr N , Bode C and Duerschmied D (2017) The effects of serotonin in immune cells. Frontiers in Cardiovascular Medicine 4, 48. DOI: 10.3389/fcvm.2017.00048. 28775986 PMC5517399

[ref96] Hibicke M , Landry AN , Kramer HM , Talman ZK and Nichols CD (2020) Psychedelics, but not ketamine, produce persistent antidepressant-like effects in a rodent experimental system for the study of depression. ACS Chemical Neuroscience 11(6), 864–871. DOI: 10.1021/acschemneuro.9b00493. 32133835

[ref97] Hinkle JT , Graziosi M , Nayak SM and Yaden DB (2024) Adverse events in studies of classic psychedelics: a systematic review and meta-analysis. JAMA Psychiatry 81(12), 1225–1235. DOI: 10.1001/jamapsychiatry.2024.2546. 39230883 PMC11375525

[ref98] Holze F , Caluori TV , Vizeli P and Liechti ME (2022) Safety pharmacology of acute LSD administration in healthy subjects. Psychopharmacology (Berl) 239(6), 1893–1905. DOI: 10.1007/s00213-021-05978-6. 34515824 PMC9166834

[ref99] Holze F , Gasser P , Müller F , Dolder PC and Liechti ME (2023) Lysergic acid diethylamide-assisted therapy in patients with anxiety with and without a life-threatening illness: a randomized, double-blind, placebo-controlled phase II study. Biological Psychiatry 93(3), 215–223. DOI: 10.1016/j.biopsych.2022.08.025. 36266118

[ref100] Holze F , Singh N , Liechti ME and D’Souza DC (2024) Serotonergic psychedelics: a comparative review of efficacy, safety, pharmacokinetics, and binding profile. Biological Psychiatry: Cognitive Neuroscience and Neuroimaging 9(5), 472–489. DOI: 10.1016/j.bpsc.2024.01.007. 38301886

[ref101] Huang J , Pham M , Panenka WJ , Honer WG and Barr AM (2022) Chronic treatment with psilocybin decreases changes in body weight in a rodent model of obesity. Frontiers in Psychiatry 13, 891512. DOI: 10.3389/fpsyt.2022.891512. 35664477 PMC9157591

[ref103] Hutten NRPW , Mason NL , Dolder PC , Theunissen EL , Holze F , Liechti ME , Feilding A , Ramaekers JG and Kuypers KPC (2020a) Mood and cognition after administration of low LSD doses in healthy volunteers: a placebo controlled dose-effect finding study. European Neuropsychopharmacology 41, 81–91. DOI: 10.1016/j.euroneuro.2020.10.002. 33082016

[ref102] Hutten NRPW , Mason NL , Dolder PC , Theunissen EL , Holze F , Liechti ME , Varghese N , Eckert A , Feilding A , Ramaekers JG and Kuypers KPC (2020b) Low doses of LSD acutely increase BDNF blood plasma levels in healthy volunteers. ACS Pharmacology & Translational Science 4(2), 461–466. DOI: 10.1021/acsptsci.0c00099. 33860175 PMC8033605

[ref104] Hysek CM , Schmid Y , Simmler LD , Domes G , Heinrichs M , Eisenegger C , Preller KH , Quednow BB and Liechti ME (2014) MDMA enhances emotional empathy and prosocial behavior. Social Cognitive and Affective Neuroscience 9(11), 1645–1652. DOI: 10.1093/scan/nst161. 24097374 PMC4221206

[ref105] Inserra A , Campanale A , Rezai T , Romualdi P and Rubino T (2024) Epigenetic mechanisms of rapid-acting antidepressants. Translational Psychiatry 14(1), 359. DOI: 10.1038/s41398-024-03055-y. 39231927 PMC11375021

[ref106] Inserra A , De Gregorio D and Gobbi G (2021) Psychedelics in psychiatry: neuroplastic, immunomodulatory, and neurotransmitter mechanisms. Pharmacological Reviews 73(1), 202–277. DOI: 10.1124/pharmrev.120.000056. 33328244

[ref107] Jardim AV , Jardim DV , Chaves BR , Steglich M , Ot’alora GM , Mithoefer MC , da Silveira DX , Tófoli LF , Ribeiro S , Matthews R , Doblin R and Schenberg EE (2021) 3,4-methylenedioxymethamphetamine (MDMA)-assisted psychotherapy for victims of sexual abuse with severe post-traumatic stress disorder: an open label pilot study in Brazil. Brazilian Journal of Psychiatry 43(2), 181–185. DOI: 10.1590/1516-4446-2020-0980. 32638920 PMC8023155

[ref108] Jerome L , Feduccia AA , Wang JB , Hamilton S , Yazar-Klosinski B , Emerson A , Mithoefer MC and Doblin R (2020) Long-term follow-up outcomes of MDMA-assisted psychotherapy for treatment of PTSD: a longitudinal pooled analysis of six phase 2 trials. Psychopharmacology (Berl) 237(8), 2485–2497. DOI: 10.1007/s00213-020-05548-2. 32500209 PMC7351848

[ref109] Johansen PØ. and Krebs TS (2015) Psychedelics not linked to mental health problems or suicidal behavior: a population study. Journal of Psychopharmacology 29(3), 270–279. DOI: 10.1177/0269881114568039. 25744618

[ref110] Johnson M , Richards W and Griffiths R (2008) Human hallucinogen research: guidelines for safety. Journal of Psychopharmacology 22(6), 603–620. DOI: 10.1177/0269881108093587. 18593734 PMC3056407

[ref111] Johnson MW , Garcia-Romeu A , Cosimano MP and Griffiths RR (2014) Pilot study of the 5-HT2AR agonist psilocybin in the treatment of tobacco addiction. Journal of Psychopharmacology 28(11), 983–992. DOI: 10.1177/0269881114548296. 25213996 PMC4286320

[ref112] Johnson MW , Garcia-Romeu A and Griffiths RR (2017) Long-term follow-up of psilocybin-facilitated smoking cessation. American Journal of Drug and Alcohol Abuse 43(1), 55–60. DOI: 10.3109/00952990.2016.1170135. 27441452 PMC5641975

[ref113] Johnson MW , Griffiths RR , Hendricks PS and Henningfield JE (2018) The abuse potential of medical psilocybin according to the 8 factors of the controlled substances act. Neuropharmacology 142, 143–166. DOI: 10.1016/j.neuropharm.2018.05.012. 29753748 PMC6791528

[ref114] Johnson MW , Hendricks PS , Barrett FS and Griffiths RR (2019) Classic psychedelics: an integrative review of epidemiology, therapeutics, mystical experience, and brain network function. Pharmacology & Therapeutics 197, 83–102. DOI: 10.1016/j.pharmthera.2018.11.010. 30521880

[ref115] Kamilar-Britt P and Bedi G (2015) The prosocial effects of 3,4-methylenedioxymethamphetamine (MDMA): controlled studies in humans and laboratory animals. Neuroscience & Biobehavioral Reviews 57, 433–446. DOI: 10.1016/j.neubiorev.2015.08.016. 26408071 PMC4678620

[ref116] Kim K , Che T , Panova O , DiBerto JF , Lyu J , Krumm BE , Wacker D , Robertson MJ , Seven AB , Nichols DE , Shoichet BK , Skiniotis G and Roth BL (2020) Structure of a hallucinogen-activated gq-coupled 5-HT_2A_ serotonin receptor. Cell 182(6), 1574–1588.e19. DOI: 10.1016/j.cell.2020.08.024. 32946782 PMC7593816

[ref117] Klaiber A , Humbert-Droz M , Ley L , Schmid Y and Liechti ME (2024) Safety pharmacology of acute mescaline administration in healthy participants. British Journal of Clinical Pharmacology. DOI: 10.1111/bcp.16349. 39587436

[ref118] Knudsen GM (2023) Sustained effects of single doses of classical psychedelics in humans. Neuropsychopharmacology 48(1), 145–150. DOI: 10.1038/s41386-022-01361-x. 35729252 PMC9700827

[ref269] Kopra EI , Penttinen J , Rucker JJ and Copeland CS (2025) Psychedelic-Related deaths in England, Wales and Northern Ireland (1997-2022). Progress in Neuro-Psychopharmacology & Biological Psychiatry 136, 111177. DOI: 10.1016/j.pnpbp.2024.111177. 39437962

[ref120] Korthuis PT , Hoffman K , Wilson-Poe AR , Luoma JB , Bazinet A , Pertl K , Morgan DL , Cook RR , Bielavitz S , Myers R , Wolf RC , McCarty D and Stauffer CS (2024) Developing the open psychedelic evaluation nexus consensus measures for assessment of supervised psilocybin services: an e-delphi study. Journal of Psychopharmacology 38(8), 761–768. DOI: 10.1177/02698811241257839. 38888164

[ref121] Kraehenmann R , Preller KH , Scheidegger M , Pokorny T , Bosch OG , Seifritz E and Vollenweider FX (2015) Psilocybin-induced decrease in amygdala reactivity correlates with enhanced positive mood in healthy volunteers. Biological Psychiatry 78(8), 572–581. DOI: 10.1016/j.biopsych.2014.04.010. 24882567

[ref122] Laabi S , LeMmon C , Vogel C , Chacon M and Jimenez VM Jr (2024) 2024Deciphering psilocybin: cytotoxicity, anti-inflammatory effects, and mechanistic insights. International Immunopharmacology 130, 111753. DOI: 10.1016/j.intimp.2024.111753. 38401463

[ref270] Lake S and Lucas P (2024) Co-Use of psychedelics with other substances: Findings from the global psychedelic survey. Journal of Psychopharmacology 28, 2698811241292956. DOI: 10.1177/02698811241292956. 39468747

[ref124] Lebedev AV , Kaelen M , Lövdén M , Nilsson J , Feilding A , Nutt DJ and Carhart-Harris RL (2016) LSD-induced entropic brain activity predicts subsequent personality change. Human Brain Mapping 37(9), 3203–3213. DOI: 10.1002/hbm.23234. 27151536 PMC6867426

[ref125] Lehmann ED , Thom CH and Croft DN (1995) Delayed severe rhabdomyolysis after taking ‘ecstasy’. Postgraduate Medical Journal 71(833), 186–187. DOI: 10.1136/pgmj.71.833.186-a. PMC2398185 7746785

[ref126] Lewis EC , Jaeger A , Girn M , Omene E , Brendle M and Argento E (2024) Exploring psychedelic-assisted therapy in the treatment of functional seizures: a review of underlying mechanisms and associated brain networks. Journal of Psychopharmacology 38(5), 407–416. DOI: 10.1177/02698811241248395. 38654554 PMC11102649

[ref127] Liao C , Dua AN , Wojtasiewicz C , Liston C and Kwan AC (2025) Structural neural plasticity evoked by rapid-acting antidepressant interventions. Nature Reviews Neurosciences 26(2), 101–114. DOI: 10.1038/s41583-024-00876-0. PMC11892022 39558048

[ref128] Lima da Cruz RV , Moulin TC , Petiz LL and Leão RN (2018) A single dose of 5-MeO-DMT stimulates cell proliferation, neuronal survivability, morphological and functional changes in adult mice ventral dentate gyrus. Frontiers in Molecular Neuroscience 11, 312. DOI: 10.3389/fnmol.2018.00312. 30233313 PMC6131656

[ref129] Liu F , Wu J , Gong Y , Wang P , Zhu L , Tong L , Chen X , Ling Y and Huang C (2017a) Harmine produces antidepressant-like effects via restoration of astrocytic functions. Progress in Neuro-Psychopharmacology & Biological Psychiatry 79(Pt B), 258–267. DOI: 10.1016/j.pnpbp.2017.06.012. 28625859

[ref130] Liu X , Li M , Tan S , Wang C , Fan S and Huang C (2017b) Harmine is an inflammatory inhibitor through the suppression of NF-κB signaling. Biochemical and Biophysical Research Communications 489(3), 332–338. DOI: 10.1016/j.bbrc.2017.05.126. 28551404

[ref131] Low ZXB , Ng WS , Lim ESY , Goh BH and Kumari Y (2025) The immunomodulatory effects of classical psychedelics: a systematic review of preclinical studies. Progress in Neuro-Psychopharmacology & Biological Psychiatry 136, 111139. DOI: 10.1016/j.pnpbp.2024.111139. 39251080

[ref132] Lukasiewicz K , Baker JJ , Zuo Y and Lu J (2021) Serotonergic psychedelics in neural plasticity. Frontiers in Molecular Neuroscience 14, 748359. DOI: 10.3389/fnmol.2021.748359. 34712118 PMC8545892

[ref133] Luoma JB , Chwyl C , Bathje GJ , Davis AK and Lancelotta R (2020) A meta-analysis of placebo-controlled trials of psychedelic-assisted therapy. Journal of Psychoactive Drugs 52(4), 289–299. DOI: 10.1080/02791072.2020.1769878. 32529966 PMC7736164

[ref134] Ly C , Greb AC , Cameron LP , Wong JM , Barragan EV , Wilson PC , Burbach KF , Soltanzadeh Zarandi S , Sood A , Paddy MR , Duim WC , Dennis MY , McAllister AK , Ori-McKenney KM , Gray JA and Olson DE (2018) Psychedelics promote structural and functional neural plasticity. Cell Reports 23(11), 3170–3182. DOI: 10.1016/j.celrep.2018.05.022. 29898390 PMC6082376

[ref135] Ma Y , Li W , Yao Q , Liu Y , Yu J , Zang L , Wang S , Zhou L , Wen S , Luo Y , Li W and Niu X (2024) Harmine ameliorates CCl_4_-induced acute liver injury through suppression of autophagy and inflammation. International Immunopharmacology 129, 111538. DOI: 10.1016/j.intimp.2024.111538. 38306830

[ref136] Madsen MK , Fisher PM , Burmester D , Dyssegaard A , Stenbæk DS , Kristiansen S , Johansen SS , Lehel S , Linnet K , Svarer C , Erritzoe D , Ozenne B and Knudsen GM (2019) Psychedelic effects of psilocybin correlate with serotonin 2A receptor occupancy and plasma psilocin levels. Neuropsychopharmacology 44(7), 1328–1334. DOI: 10.1038/s41386-019-0324-9. 30685771 PMC6785028

[ref137] Marí-Sanchis A , Burgos-Balmaseda J and Hidalgo-Borrajo R (2022) Eating disorders in sport. Update and proposal for an integrated approach. Endocrinology, Diabetes and Nutrition (Engl Ed) 69(2), 131–143. DOI: 10.1016/j.endien.2022.02.016. 35256056

[ref138] Marton S , González B , Rodríguez-Bottero S , Miquel E , Martínez-Palma L , Pazos M , Prieto JP , Rodríguez P , Sames D , Seoane G , Scorza C , Cassina P and Carrera I (2019) 2019Ibogaine administration modifies GDNF and BDNF expression in brain regions involved in Mesocorticolimbic and nigral dopaminergic circuits. Frontiers in Pharmacology 10, 193. DOI: 10.3389/fphar.2019.00193. 30890941 PMC6411846

[ref139] Mason NL , Kuypers KPC , Müller F , Reckweg J , Tse DHY , Toennes SW , Hutten NRPW , Jansen JFA , Stiers P , Feilding A and Ramaekers JG (2020) Me, myself, bye: regional alterations in glutamate and the experience of ego dissolution with psilocybin. Neuropsychopharmacology 45(12), 2003–2011. DOI: 10.1038/s41386-020-0718-8. 32446245 PMC7547711

[ref141] Mason NL , Szabo A , Kuypers KPC , Mallaroni PA , de la Torre Fornell R , Reckweg JT , Tse DHY , Hutten NRPW , Feilding A and Ramaekers JG (2023) Psilocybin induces acute and persisting alterations in immune status in healthy volunteers: an experimental, placebo-controlled study. Brain, Behavior, and Immunity 114, 299–310. DOI: 10.1016/j.bbi.2023.09.004. 37689275

[ref142] McClure-Begley TD and Roth BL (2022) The promises and perils of psychedelic pharmacology for psychiatry. Nature Reviews Drug Discovery 21(6), 463–473. DOI: 10.1038/s41573-022-00421-7. 35301459

[ref143] McDonald C , Losty C and MacCarthy R (2023) An investigation of the psychological status of amateur athletes before and after a triathlon competition. European Journal of Sport Sciences 2(3), 14–20. DOI: 10.24018/ejsport.2023.2.3.83.

[ref144] McIntyre RS , Alsuwaidan M , Baune BT , Berk M , Demyttenaere K , Goldberg JF , Gorwood P , Ho R , Kasper S , Kennedy SH , Ly-Uson J , Mansur RB , McAllister-Williams RH , Murrough JW , Nemeroff CB , Nierenberg AA , Rosenblat JD , Sanacora G , Schatzberg AF , Shelton R , Stahl SM , Trivedi MH , Vieta E , Vinberg M , Williams N , Young AH and Maj M (2023) Treatment-resistant depression: definition, prevalence, detection, management, and investigational interventions. World Psychiatry 22(3), 394–412. DOI: 10.1002/wps.21120. 37713549 PMC10503923

[ref145] Melani A , Bonaso M , Biso L , Zucchini B , Conversano C and Scarselli M (2025) Uncovering psychedelics: from neural circuits to therapeutic applications. Pharmaceuticals (Basel) 18(1), 130. DOI: 10.3390/ph18010130. 39861191 PMC11769142

[ref146] Mendes FR , Costa CDS , Wiltenburg VD , Morales-Lima G , Fernandes JB and Filev R (2022) Classic and non-classic psychedelics for substance use disorder: a review of their historic, past and current research. Addiction Neuroscience 3, 100025. DOI: 10.1016/j.addicn.2022.100025.

[ref147] Mertens LJ , Wall MB , Roseman L , Demetriou L , Nutt DJ and Carhart-Harris RL (2020) Therapeutic mechanisms of psilocybin: changes in amygdala and prefrontal functional connectivity during emotional processing after psilocybin for treatment-resistant depression. Journal of Psychopharmacology 34(2), 167–180. DOI: 10.1177/0269881119895520. 31941394

[ref148] Mitchell JM and Anderson BT (2024) Psychedelic therapies reconsidered: compounds, clinical indications, and cautious optimism. Neuropsychopharmacology 49(1), 96–103. DOI: 10.1038/s41386-023-01656-7. 37479859 PMC10700471

[ref149] Mitchell JM , Bogenschutz M , Lilienstein A , Harrison C , Kleiman S , Parker-Guilbert K , Ot’alora GM , Garas W , Paleos C , Gorman I , Nicholas C , Mithoefer M , Carlin S , Poulter B , Mithoefer A , Quevedo S , Wells G , Klaire SS , van der Kolk B , Tzarfaty K , Amiaz R , Worthy R , Shannon S , Woolley JD , Marta C , Gelfand Y , Hapke E , Amar S , Wallach Y , Brown R , Hamilton S , Wang JB , Coker A , Matthews R , de Boer A , Yazar-Klosinski B , Emerson A and Doblin R (2021) MDMA-assisted therapy for severe PTSD: a randomized, double-blind, placebo-controlled phase 3 study. Nature Medicine 27(6), 1025–1033. DOI: 10.1038/s41591-021-01336-3. PMC8205851 33972795

[ref150] Mitchell JM , Ot’alora GM , van der Kolk B , Shannon S , Bogenschutz M , Gelfand Y , Paleos C , Nicholas CR , Quevedo S , Balliett B , Hamilton S , Mithoefer M , Kleiman S , Parker-Guilbert K , Tzarfaty K , Harrison C , de Boer A , Doblin R Yazar-Klosinski B and MAPP2 Study Collaborator Group (2023) MDMA-assisted therapy for moderate to severe PTSD: a randomized, placebo-controlled phase 3 trial. Nature Medicine 29(10), 2473–2480. DOI: 10.1038/s41591-023-02565-4 . PMC10579091 37709999

[ref151] Mithoefer MC , Feduccia AA , Jerome L , Mithoefer A , Wagner M , Walsh Z , Hamilton S , Yazar-Klosinski B , Emerson A and Doblin R (2019) MDMA-assisted psychotherapy for treatment of PTSD: study design and rationale for phase 3 trials based on pooled analysis of six phase 2 randomized controlled trials. Psychopharmacology (Berl) 236(9), 2735–2745. DOI: 10.1007/s00213-019-05249-5. 31065731 PMC6695343

[ref152] Mithoefer MC , Mithoefer AT , Feduccia AA , Jerome L , Wagner M , Wymer J , Holland J , Hamilton S , Yazar-Klosinski B , Emerson A and Doblin R (2018) 3,4-methylenedioxymethamphetamine (MDMA)-assisted psychotherapy for post-traumatic stress disorder in military veterans, firefighters, and police officers: a randomised, double-blind, dose-response, phase 2 clinical trial. Lancet Psychiatry 5(6), 486–497. DOI: 10.1016/S2215-0366(18)30135-4. 29728331

[ref153] Mithoefer MC , Wagner MT , Mithoefer AT , Jerome L and Doblin R (2011) The safety and efficacy of {+/-}3,4-methylenedioxymethamphetamine-assisted psychotherapy in subjects with chronic, treatment-resistant posttraumatic stress disorder: the first randomized controlled pilot study. Journal of Psychopharmacology 25(4), 439–452. DOI: 10.1177/0269881110378371. 20643699 PMC3122379

[ref154] Mithoefer MC , Wagner MT , Mithoefer AT , Jerome L , Martin SF , Yazar-Klosinski B , Michel Y , Brewerton TD and Doblin R (2013) Durability of improvement in post-traumatic stress disorder symptoms and absence of harmful effects or drug dependency after 3,4-methylenedioxymethamphetamine-assisted psychotherapy: a prospective long-term follow-up study. Journal of Psychopharmacology 27(1), 28–39. DOI: 10.1177/0269881112456611. 23172889 PMC3573678

[ref155] Moliner R , Girych M , Brunello CA , Kovaleva V , Biojone C , Enkavi G , Antenucci L , Kot EF , Goncharuk SA , Kaurinkoski K , Kuutti M , Fred SM , Elsilä LV , Sakson S , Cannarozzo C , Diniz CRAF , Seiffert N , Rubiolo A , Haapaniemi H , Meshi E , Nagaeva E , Öhman T , Róg T , Kankuri E , Vilar M , Varjosalo M , Korpi ER , Permi P , Mineev KS , Saarma M , Vattulainen I , Casarotto PC and Castrén E (2023) Psychedelics promote plasticity by directly binding to BDNF receptor trkB. Nature Neuroscience 26(6), 1032–1041. DOI: 10.1038/s41593-023-01316-5. 37280397 PMC10244169

[ref156] Morales-Garcia JA , Calleja-Conde J , Lopez-Moreno JA , Alonso-Gil S , Sanz-SanCristobal M , Riba J and Perez-Castillo A (2020) N, N-dimethyltryptamine compound found in the hallucinogenic tea ayahuasca, regulates adult neurogenesis in vitro and in vivo. Translational Psychiatry 10(1), 331. DOI: 10.1038/s41398-020-01011-0. 32989216 PMC7522265

[ref157] Morris AD (2015) Improving pharmaceutical care for athletes: a proposed assessment tool and useful resources. Canadian Pharmacists Journal 148(6), 305–307. DOI: 10.1177/1715163515607309. 26600821 PMC4637850

[ref158] Munier JJ , Pank JT , Severino A , Wang H , Zhang P , Vergnes L and Reue K (2022) Simultaneous monitoring of mouse grip strength, force profile, and cumulative force profile distinguishes muscle physiology following surgical, pharmacologic and diet interventions. Scientific Reports 12(1), 16428. DOI: 10.1038/s41598-022-20665-y. 36180720 PMC9525296

[ref159] Murphy RJ , Muthukumaraswamy S and de Wit H (2024) Microdosing psychedelics: current evidence from controlled studies. Biological Psychiatry: Cognitive Neuroscience and Neuroimaging 9(5), 500–511. DOI: 10.1016/j.bpsc.2024.01.002. 38280630

[ref160] Nardai S , László M , Szabó A , Alpár A , Hanics J , Zahola P , Merkely B , Frecska E and Nagy Z (2020) N, N-dimethyltryptamine reduces infarct size and improves functional recovery following transient focal brain ischemia in rats. Experimental Neurology 327, 113245. DOI: 10.1016/j.expneurol.2020.113245. 32067950

[ref161] Nau F Jr , Miller J , Saravia J , Ahlert T , Yu B , Happel KI , Cormier SA and Nichols CD (2015) Serotonin 5-HT2 receptor activation prevents allergic asthma in a mouse model. American Journal of Physiology-Lung Cellular and Molecular Physiology 308(2), L191–8. DOI: 10.1152/ajplung.00138.2013. 25416380 PMC4338939

[ref162] Nau F Jr , Yu B , Martin D and Nichols CD (2013) Serotonin 5-HT2A receptor activation blocks TNF-α mediated inflammation in vivo. PLoS One 8(10), e75426. DOI: 10.1371/journal.pone.0075426. 24098382 PMC3788795

[ref163] Neumann J , Dhein S , Kirchhefer U , Hofmann B and Gergs U (2024) Effects of hallucinogenic drugs on the human heart. Frontiers in Pharmacology 15, 1334218. DOI: 10.3389/fphar.2024.1334218. 38370480 PMC10869618

[ref164] Nicholas CR , Wang JB , Coker A , Mitchell JM , Klaire SS , Yazar-Klosinski B , Emerson A , Brown RT and Doblin R (2022) The effects of MDMA-assisted therapy on alcohol and substance use in a phase 3 trial for treatment of severe PTSD. Drug and Alcohol Dependence 233, 109356. DOI: 10.1016/j.drugalcdep.2022.109356. 35286849 PMC9750500

[ref165] Nichols DE (2016) Psychedelics. Pharmacological Reviews 68(2), 264–355. DOI: 10.1124/pr.115.011478. 26841800 PMC4813425

[ref166] Niu X , Yao Q , Li W , Zang L , Li W , Zhao J , Liu F and Zhi W (2019) Harmine mitigates LPS-induced acute kidney injury through inhibition of the TLR4-NF-κB/NLRP3 inflammasome signalling pathway in mice. European Journal of Pharmacology 849, 160–169. DOI: 10.1016/j.ejphar.2019.01.062. 30716318

[ref167] Nkadimeng SM , Steinmann CML and Eloff JN (2021) Anti-inflammatory effects of four psilocybin-containing magic mushroom water extracts in vitro on 15-lipoxygenase activity and on lipopolysaccharide-induced cyclooxygenase-2 and inflammatory cytokines in human U937 macrophage cells. Journal of Inflammation Research 14, 3729–3738. DOI: 10.2147/JIR.S317182. 34385833 PMC8352634

[ref168] Nutt D and Carhart-Harris R (2021) The current status of psychedelics in psychiatry. JAMA Psychiatry 78(2), 121–122. DOI: 10.1001/jamapsychiatry.2020.2171. 32725172

[ref169] Nutt D , Crome I and Young AH (2024) Is it now time to prepare psychiatry for a psychedelic future? British Journal of Psychiatry 225(2), 308–310. DOI: 10.1192/bjp.2024.76. 38764044

[ref171] Odland AU , Kristensen JL and Andreasen JT (2022) Animal behavior in psychedelic research. Pharmacological Reviews 74(4), 1176–1205. DOI: 10.1124/pharmrev.122.000590. 36180111

[ref172] Oehen P , Traber R , Widmer V and Schnyder U (2013) A randomized, controlled pilot study of MDMA (± 3,4-methylenedioxymethamphetamine)-assisted psychotherapy for treatment of resistant, chronic post-traumatic stress disorder (PTSD). Journal of Psychopharmacology 27(1), 40–52. DOI: 10.1177/0269881112464827. 23118021

[ref173] Olson DE (2022) Biochemical mechanisms underlying psychedelic-induced neuroplasticity. Biochemistry 61(3), 127–136. DOI: 10.1021/acs.biochem.1c00812. 35060714 PMC9004607

[ref174] Osmond H (1957) A review of the clinical effects of psychotomimetic agents. Annals of the New York Academy of Sciences 66(3), 418–434. DOI: 10.1111/j.1749-6632.1957.tb40738.x. 13425232

[ref175] Ot’alora GM , Grigsby J , Poulter B , Van Derveer JW 3rd, Giron SG , Jerome L , Feduccia AA , Hamilton S , Yazar-Klosinski B , Emerson A , Mithoefer MC and Doblin R (2018) 3,4-methylenedioxymethamphetamine-assisted psychotherapy for treatment of chronic posttraumatic stress disorder: a randomized phase 2 controlled trial. Journal of Psychopharmacology 32(12), 1295–1307. DOI: 10.1177/0269881118806297. 30371148 PMC6247454

[ref176] Pagni BA , Petridis PD , Podrebarac SK , Grinband J , Claus ED and Bogenschutz MP (2024) Psilocybin-induced changes in neural reactivity to alcohol and emotional cues in patients with alcohol use disorder: an fMRI pilot study. Scientific Reports 14(1), 3159. DOI: 10.1038/s41598-024-52967-8. 38326432 PMC10850478

[ref177] Palhano-Fontes F , Andrade KC , Tofoli LF , Santos AC , Crippa JA , Hallak JE , Ribeiro S and de Araujo DB (2015) The psychedelic state induced by ayahuasca modulates the activity and connectivity of the default mode network. PLoS One 10(2), e0118143. DOI: 10.1371/journal.pone.0118143. 25693169 PMC4334486

[ref178] Palhano-Fontes F , Barreto D , Onias H , Andrade KC , Novaes MM , Pessoa JA , Mota-Rolim SA , Osório FL , Sanches R , Dos Santos RG , Tófoli LF , de Oliveira Silveira G , Yonamine M , Riba J , Santos FR , Silva-Junior AA , Alchieri JC , Galvão-Coelho NL , Lobão-Soares B , Hallak JEC , Arcoverde E , Maia-de-Oliveira JP and Araújo DB (2019) Rapid antidepressant effects of the psychedelic ayahuasca in treatment-resistant depression: a randomized placebo-controlled trial. Psychological Medicine 49(4), 655–663. DOI: 10.1017/S0033291718001356. 29903051 PMC6378413

[ref179] Peck SK , Shao S , Gruen T , Yang K , Babakanian A , Trim J , Finn DM and Kaye WH (2023) Psilocybin therapy for females with anorexia nervosa: a phase 1, open-label feasibility study. Nature Medicine 29(8), 1947–1953. DOI: 10.1038/s41591-023-02455-9. PMC10427429 37488291

[ref180] Peill J , Marguilho M , Erritzoe D , Barba T , Greenway KT , Rosas F , Timmermann C and Carhart-Harris R (2024) Psychedelics and the ‘inner healer’: myth or mechanism? Journal of Psychopharmacology 38(5), 417–424. DOI: 10.1177/02698811241239206. 38605658 PMC11102647

[ref181] Perkins D , Pagni BA , Sarris J , Barbosa PCR and Chenhall R (2022) Changes in mental health, wellbeing and personality following ayahuasca consumption: results of a naturalistic longitudinal study. Frontiers in Pharmacology 13, 884703. DOI: 10.3389/fphar.2022.884703. 36386199 PMC9643165

[ref183] Polito V and Stevenson RJ (2019) A systematic study of microdosing psychedelics. PLoS One 14(2), e0211023. DOI: 10.1371/journal.pone.0211023. 30726251 PMC6364961

[ref184] Ponte L , Jerome L , Hamilton S , Mithoefer MC , Yazar-Klosinski BB , Vermetten E and Feduccia AA (2021) Sleep quality improvements after MDMA-assisted psychotherapy for the treatment of posttraumatic stress disorder. Journal of Traumatic Stress 34(4), 851–863. DOI: 10.1002/jts.22696. 34114250 PMC8453707

[ref185] Preller KH , Burt JB , Ji JL , Schleifer CH , Adkinson BD , Stämpfli P , Seifritz E , Repovs G , Krystal JH , Murray JD , Vollenweider FX and Anticevic A (2018) Changes in global and thalamic brain connectivity in LSD-induced altered states of consciousness are attributable to the 5-HT2A receptor. Elife 7, e35082. DOI: 10.7554/eLife.35082. 30355445 PMC6202055

[ref186] Preller KH , Duerler P , Burt JB , Ji JL , Adkinson B , Stämpfli P , Seifritz E , Repovš G , Krystal JH , Murray JD , Anticevic A and Vollenweider FX (2020) Psilocybin induces time-dependent changes in global functional connectivity. Biological Psychiatry 88(2), 197–207. DOI: 10.1016/j.biopsych.2019.12.027. 32111343

[ref187] Rakoczy RJ , Runge GN , Sen AK , Sandoval O , Wells HG , Nguyen Q , Roberts BR , Sciortino JH , Gibbons WJ Jr , Friedberg LM , Jones JA and McMurray MS (2024) Pharmacological and behavioural effects of tryptamines present in psilocybin-containing mushrooms. British Journal of Pharmacology 181(19), 3627–3641. DOI: 10.1111/bph.16466. 38825326

[ref188] Ramaekers JG , Hutten N , Mason NL , Dolder P , Theunissen EL , Holze F , Liechti ME , Feilding A and Kuypers KP (2021) A low dose of lysergic acid diethylamide decreases pain perception in healthy volunteers. Journal of Psychopharmacology 35(4), 398–405. DOI: 10.1177/0269881120940937. 32842825 PMC8054163

[ref189] Raote I , Bhattacharya A and Panicker MM (2007) Serotonin 2A (5-HT_2A_) receptor function: ligand-dependent mechanisms and pathways. In Chattopadhyay A (ed), Serotonin receptors in neurobiology. Boca Raton (FL): CRC Press/Taylor & Francis, Chapter 6, https://www.ncbi.nlm.nih.gov/books/NBK1853/ 21204452

[ref190] Ray TS (2010) Psychedelics and the human receptorome. PLoS One 5(2), e9019. DOI: 10.1371/journal.pone.0009019. 20126400 PMC2814854

[ref193] Reardon CL (2016) The sports psychiatrist and psychiatric medication. International Review of Psychiatry 28(6), 606–613. DOI: 10.1080/09540261.2016.1190691. 27329699

[ref191] Reardon CL and Creado S (2016) Psychiatric medication preferences of sports psychiatrists. Physician and Sportsmedicine 44(4), 397–402. DOI: 10.1080/00913847.2016.1216719. 27463033

[ref192] Reardon CL , Hainline B , Aron CM , Baron D , Baum AL , Bindra A , Budgett R , Campriani N , Castaldelli-Maia JM , Currie A , Derevensky JL , Glick ID , Gorczynski P , Gouttebarge V , Grandner MA , Han DH , McDuff D , Mountjoy M , Polat A , Purcell R , Putukian M , Rice S , Sills A , Stull T , Swartz L , Zhu LJ and Engebretsen L (2019) Mental health in elite athletes: International Olympic Committee consensus statement (2019). British Journal of Sports Medicine 53(11), 667–699. DOI: 10.1136/bjsports-2019-100715. 31097450

[ref194] Reiff CM , Richman EE , Nemeroff CB , Carpenter LL , Widge AS , Rodriguez CI , Kalin NH and McDonald WM (2020) The work group on biomarkers and novel treatments, a division of the American Psychiatric Association Council of Research, 2020 psychedelics and psychedelic-assisted psychotherapy. American Journal of Psychiatry 177(5), 391–410. DOI: 10.1176/appi.ajp.2019.19010035. 32098487

[ref195] Rhee TG , Davoudian PA , Sanacora G and Wilkinson ST (2023) Psychedelic renaissance: revitalized potential therapies for psychiatric disorders. Drug Discovery Today 28(12), 103818. DOI: 10.1016/j.drudis.2023.103818. 37925136

[ref196] Riba J , Valle M , Urbano G , Yritia M , Morte A and Barbanoj MJ (2003) Human pharmacology of ayahuasca: subjective and cardiovascular effects, monoamine metabolite excretion, and pharmacokinetics. Journal of Pharmacology and Experimental Therapeutics 306(1), 73–83. DOI: 10.1124/jpet.103.049882. 12660312

[ref197] Romeo B , Kervadec E , Fauvel B , Strika-Bruneau L , Amirouche A , Verroust V , Piolino P and Benyamina A (2024) Safety and risk assessment of psychedelic psychotherapy: a meta-analysis and systematic review. Psychiatry Research 335, 115880. DOI: 10.1016/j.psychres.2024.115880. 38579460

[ref198] Roseman L , Demetriou L , Wall MB , Nutt DJ and Carhart-Harris RL (2018) Increased amygdala responses to emotional faces after psilocybin for treatment-resistant depression. Neuropharmacology 142, 263–269. DOI: 10.1016/j.neuropharm.2017.12.041. 29288686

[ref199] Ross S , Bossis A , Guss J , Agin-Liebes G , Malone T , Cohen B , Mennenga SE , Belser A , Kalliontzi K , Babb J , Su Z , Corby P and Schmidt BL (2016) Rapid and sustained symptom reduction following psilocybin treatment for anxiety and depression in patients with life-threatening cancer: a randomized controlled trial. Journal of Psychopharmacology 30(12), 1165–1180. DOI: 10.1177/0269881116675512. 27909164 PMC5367551

[ref200] Rusyniak DE , Tandy SL , Hekmatyar SK , Mills E , Smith DJ , Bansal N , MacLellan D , Harper ME and Sprague JE (2005) The role of mitochondrial uncoupling in 3,4-methylenedioxymethamphetamine-mediated skeletal muscle hyperthermia and rhabdomyolysis. Journal of Pharmacology and Experimental Therapeutics 313(2), 629–639. DOI: 10.1124/jpet.104.079236. 15644431

[ref201] Sabé M , Sulstarova A , Glangetas A , De Pieri M , Mallet L , Curtis L , Richard-Lepouriel H , Penzenstadler L , Seragnoli F , Thorens G , Zullino D , Preller K , Böge K , Leucht S , Correll CU , Solmi M , Kaiser S and Kirschner M (2025) Reconsidering evidence for psychedelic-induced psychosis: an overview of reviews, a systematic review, and meta-analysis of human studies. Molecular Psychiatry 30(3), 1223–1255. DOI:10.1038/s41380-024-02800-5. 39592825 PMC11835720

[ref202] Sanches RF , de Lima Osório F , Dos Santos RG , Macedo LR , Maia-de-Oliveira JP , Wichert-Ana L , de Araujo DB , Riba J , Crippa JA and Hallak JE (2016) Antidepressant effects of a single dose of ayahuasca in patients with recurrent depression: a SPECT study. Journal of Clinical Psychopharmacology 36(1), 77–81. DOI: 10.1097/JCP.0000000000000436. 26650973

[ref203] Sarparast A , Thomas K , Malcolm B and Stauffer CS (2022) Drug-drug interactions between psychiatric medications and MDMA or psilocybin: a systematic review. Psychopharmacology (Berl) 239(6), 1945–1976. DOI: 10.1007/s00213-022-06083-y. 35253070 PMC9177763

[ref204] Schlag AK , Aday J , Salam I , Neill JC and Nutt DJ (2022) Adverse effects of psychedelics: from anecdotes and misinformation to systematic science. Journal of Psychopharmacology 36(3), 258–272. DOI: 10.1177/02698811211069100. 35107059 PMC8905125

[ref206] Schmid Y and Liechti ME (2018) Long-lasting subjective effects of LSD in normal subjects. Psychopharmacology (Berl) 235(2), 535–545. DOI: 10.1007/s00213-017-4733-3. 28918441 PMC5813062

[ref207] Schneier FR , Feusner J , Wheaton MG , Gomez GJ , Cornejo G , Naraindas AM and Hellerstein DJ (2023) Pilot study of single-dose psilocybin for serotonin reuptake inhibitor-resistant body dysmorphic disorder. Journal of Psychiatric Research 161, 364–370. DOI: 10.1016/j.jpsychires.2023.03.031. 37004409 PMC10967229

[ref208] Screaton GR , Singer M , Cairns HS , Thrasher A , Sarner M and Cohen SL (1992) Hyperpyrexia and rhabdomyolysis after MDMA (“ecstasy”) abuse. Lancet 339(8794), 677–678. DOI: 10.1016/0140-6736(92)90834-p. 1347361

[ref209] Shafiee A , Arabzadeh Bahri R , Rafiei MA , Esmaeilpur Abianeh F , Razmara P , Jafarabady K and Amini MJ (2024) The effect of psychedelics on the level of brain-derived neurotrophic factor: a systematic review and meta-analysis. Journal of Psychopharmacology 38(5), 425–431. DOI: 10.1177/02698811241234247. 38385351

[ref210] Shahraki MR and Irani M (2014) The effects of ecstasy on liver function tests, blood glucose, and lipids profile of male rats. International Journal of High Risk Behaviors and Addiction 3(4), e21076. DOI: 10.5812/ijhrba.21076. 25741481 PMC4331654

[ref211] Shakir J , Pedicini M , Bullock BC , Hoen PW , Macias LK , Freiman J , Pletnikov MV , Tamashiro KLK and Cordner ZA (2024) Effects of psilocybin on body weight, body composition, and metabolites in male and female mice. Physiology & Behavior 284, 114627. DOI: 10.1016/j.physbeh.2024.114627. 38964565 PMC11323168

[ref212] Shan J , Khelashvili G , Mondal S , Mehler EL and Weinstein H (2012) Ligand-dependent conformations and dynamics of the serotonin 5-HT(2A) receptor determine its activation and membrane-driven oligomerization properties. PLoS Computational Biology 8(4), e1002473. DOI: 10.1371/journal.pcbi.1002473. 22532793 PMC3330085

[ref213] Shao LX , Liao C , Gregg I , Davoudian PA , Savalia NK , Delagarza K and Kwan AC (2021) Psilocybin induces rapid and persistent growth of dendritic spines in frontal cortex in vivo. Neuron 109(16), 2535–2544.e4. DOI: 10.1016/j.neuron.2021.06.008. 34228959 PMC8376772

[ref214] Shen L , Lv X , Yang X , Deng S , Liu L , Zhou J , Zhu Y and Ma H (2022) Bufotenines-loaded liposome exerts anti-inflammatory, analgesic effects and reduce gastrointestinal toxicity through altering lipid and bufotenines metabolism. Biomedicine & Pharmacotherapy 153, 113492. DOI: 10.1016/j.biopha.2022.113492. 36076586

[ref215] Siegel JS , Subramanian S , Perry D , Kay BP , Gordon EM , Laumann TO , Reneau TR , Metcalf NV , Chacko RV , Gratton C , Horan C , Krimmel SR , Shimony JS , Schweiger JA , Wong DF , Bender DA , Scheidter KM , Whiting FI , Padawer-Curry JA , Shinohara RT , Chen Y , Moser J , Yacoub E , Nelson SM , Vizioli L , Fair DA , Lenze EJ , Carhart-Harris R , Raison CL , Raichle ME , Snyder AZ , Nicol GE and Dosenbach NUF (2024) Psilocybin desynchronizes the human brain. Nature 632(8023), 131–138. DOI: 10.1038/s41586-024-07624-5. 39020167 PMC11291293

[ref216] Simon MW , Olsen HA , Hoyte CO , Black JC , Reynolds KM , Dart RC and Monte AA (2024) Clinical effects of psychedelic substances reported to United States poison centers: 2012 to 2022. Annals of Emergency Medicine 84(6), 605–618. DOI: 10.1016/j.annemergmed.2024.06.025. 39093248

[ref217] Simonsson O , Osika W , Carhart-Harris R and Hendricks PS (2021) Associations between lifetime classic psychedelic use and cardiometabolic diseases. Scientific Reports 11(1), 14427. DOI: 10.1038/s41598-021-93787-4. 34257396 PMC8277805

[ref218] Singleton SP , Wang JB , Mithoefer M , Hanlon C , George MS , Mithoefer A , Mithoefer O , Coker AR , Yazar-Klosinski B , Emerson A , Doblin R and Kuceyeski A (2023) Altered brain activity and functional connectivity after MDMA-assisted therapy for post-traumatic stress disorder. Frontiers in Psychiatry 13, 947622. DOI: 10.3389/fpsyt.2022.947622. 36713926 PMC9879604

[ref219] Skosnik PD , Sloshower J , Safi-Aghdam H , Pathania S , Syed S , Pittman B and D’Souza DC (2023) Sub-acute effects of psilocybin on EEG correlates of neural plasticity in major depression: relationship to symptoms. Journal of Psychopharmacology 37(7), 687–697. DOI: 10.1177/02698811231179800. 37392016

[ref220] Slattery DA and Cryan JF (2012) Using the rat forced swim test to assess antidepressant-like activity in rodents. Nature Protocols 7(6), 1009–1014. DOI: 10.1038/nprot.2012.044. 22555240

[ref221] Sloshower J , Skosnik PD , Safi-Aghdam H , Pathania S , Syed S , Pittman B and D’Souza DC (2023) Psilocybin-assisted therapy for major depressive disorder: an exploratory placebo-controlled, fixed-order trial. Journal of Psychopharmacology 37(7), 698–706. DOI: 10.1177/02698811231154852. 36938991

[ref222] Sloshower J , Zeifman RJ , Guss J , Krause R , Safi-Aghdam H , Pathania S , Pittman B and D’Souza DC (2024) Psychological flexibility as a mechanism of change in psilocybin-assisted therapy for major depression: results from an exploratory placebo-controlled trial. Scientific Reports 14(1), 8833. DOI: 10.1038/s41598-024-58318-x. 38632313 PMC11024097

[ref223] Smith A , Buadze A , Colangelo J and Liebrenz M (2023) A review of mental health issues in high-performance and elite-level cycling. International Journal of Sports Medicine 44(14), 1034–1042. DOI: 10.1055/a-2145-6355. 37524113

[ref224] Soto-Angona Ó. , Fortea A , Fortea L , Martínez-Ramírez M , Santamarina E , López FJG , Knudsen GM and Ona G (2024) Do classic psychedelics increase the risk of seizures? A scoping review. European Neuropsychopharmacology 85, 35–42. DOI: 10.1016/j.euroneuro.2024.05.002. 38917636

[ref225] Soto-Montenegro ML , Vaquero JJ , Arango C , Ricaurte G , García-Barreno P and Desco M (2007) Effects of MDMA on blood glucose levels and brain glucose metabolism. European Journal of Nuclear Medicine and Molecular Imaging 34(6), 916–925. DOI: 10.1007/s00259-006-0262-8. 17219137

[ref226] Sprague JE , Brutcher RE , Mills EM , Caden D and Rusyniak DE (2004) Attenuation of 3,4-methylenedioxymethamphetamine (MDMA, ecstasy)-induced rhabdomyolysis with alpha1- plus beta3-adrenoreceptor antagonists. British Journal of Pharmacology 142(4), 667–670. DOI: 10.1038/sj.bjp.0705823. 15159279 PMC1575043

[ref227] Sprague JE , Moze P , Caden D , Rusyniak DE , Holmes C , Goldstein DS and Mills EM (2005) Carvedilol reverses hyperthermia and attenuates rhabdomyolysis induced by 3,4-methylenedioxymethamphetamine (MDMA, ecstasy) in an animal model. Critical Care Medicine 33(6), 1311–1316. DOI: 10.1097/01.ccm.0000165969.29002.70. 15942349

[ref228] Stankevicius D , Ferraz-de-Paula V , Ribeiro A , Pinheiro ML , Ligeiro de Oliveira AP , Damazo AS , Lapachinske SF , Moreau RL , Tavares de Lima W and Palermo-Neto J (2012) 3,4-methylenedioxymethamphetamine (ecstasy) decreases inflammation and airway reactivity in a murine model of asthma. Neuroimmunomodulation 19(4), 209–219. DOI: 10.1159/000334098. 22441537

[ref230] Stoliker D , Novelli L , Vollenweider FX , Egan GF , Preller KH and Razi A (2024) Neural mechanisms of resting-state networks and the amygdala underlying the cognitive and emotional effects of psilocybin. Biological Psychiatry 96(1), 57–66. DOI: 10.1016/j.biopsych.2024.01.002. 38185235

[ref231] Stone MB , Yaseen ZS , Miller BJ , Richardville K , Kalaria SN and Kirsch I (2022) Response to acute monotherapy for major depressive disorder in randomized, placebo controlled trials submitted to the US Food and Drug Administration: individual participant data analysis. British Medical Journal 378, e067606. DOI: 10.1136/bmj-2021-067606. 35918097 PMC9344377

[ref229] Strand NH , Whitney M , Johnson B , Dunn T , Attanti S , Maloney J , Misra L , Gomez D , Viswanath O , Emami E and Leathem J (2025) Pain and perception: exploring psychedelics as novel therapeutic agents in chronic pain management. Current Pain and Headache Reports 29(1), 15. DOI: 10.1007/s11916-024-01353-0. 39775134

[ref232] Sue YM , Lee YL and Huang JJ (2002) Acute hyponatremia, seizure, and rhabdomyolysis after ecstasy use. Journal of Toxicology: Clinical Toxicology 40(7), 931–932. DOI: 10.1081/clt-120016964. 12507065

[ref233] Sun J , Yang X , Zhang Y , Zhang W , Lu J , Hu Q , Liu R , Zhou C and Chen C (2019) Salvinorin a attenuates early brain injury through PI3K/Akt pathway after subarachnoid hemorrhage in rat. Brain Research 1719, 64–70. DOI: 10.1016/j.brainres.2019.05.026. 31125530

[ref234] Tabaa MME , Tabaa MME , Rashad E , Elballal MS , and Elazazy O (2024) Harmine alleviated STZ-induced rat diabetic nephropathy: a potential role via regulating AMPK/Nrf2 pathway and deactivating ataxia-telangiectasia mutated (ATM) signaling. International Immunopharmacology 132, 111954. DOI: 10.1016/j.intimp.2024.111954. 38554444

[ref235] Thomas K (2024) Toxicology and pharmacological interactions of classic psychedelics, Current topics in behavioral Neurosciences. 10.1007/7854_2024_508. 39042251

[ref236] Thompson C and Szabo A (2020) Psychedelics as a novel approach to treating autoimmune conditions. Immunology Letters 228, 45–54. DOI: 10.1016/j.imlet.2020.10.001. 33035575

[ref237] Thuany M , Viljoen C , Gomes TN , Knechtle B and Scheer V (2023) Mental health in ultra-endurance runners: a systematic review. Sports Medicine 53(10), 1891–1904. DOI: 10.1007/s40279-023-01890-5. 37535248

[ref238] Tso JV and Pelliccia A (2022) Psychiatric medications and cardiovascular performance: uncommon depressing side effects. Journals of the American College of Cardiology Case Reports 4(20), 1341–1343. DOI: 10.1016/j.jaccas.2022.08.004. PMC9588446 36299654

[ref239] Vamvakopoulou IA and Nutt DJ (2024) Psychedelics: from cave art to 21st-century medicine for addiction. European Addiction Research 30(5), 302–320. DOI: 10.1159/000540062. 39321788 PMC11527458

[ref240] van der Kolk BA , Wang JB , Yehuda R , Bedrosian L , Coker AR , Harrison C , Mithoefer M , Yazar-Klosinki B , Emerson A and Doblin R (2024). Effects of MDMA-assisted therapy for PTSD on self-experience. PLoS One 19(1), e0295926. DOI: 10.1371/journal.pone.0295926. 38198456 PMC10781106

[ref241] van Oorsouw K , Toennes SW and Ramaekers JG (2022) Therapeutic effect of an ayahuasca analogue in clinically depressed patients: a longitudinal observational study. Psychopharmacology (Berl) 239(6), 1839–1852. DOI: 10.1007/s00213-021-06046-9. 35072760 PMC8785027

[ref242] Vanden Eede H , Montenij LJ , Touw DJ and Norris EM (2012) Rhabdomyolysis in MDMA intoxication: a rapid and underestimated killer. “Clean” ecstasy, a safe party drug? Journal of Emergency Medicine 42(6), 655–658. DOI: 10.1016/j.jemermed.2009.04.057. 19500935

[ref243] VanderZwaag B , Garcia-Romeu A and Garcia-Barrera MA (2024) Exploring psychedelic use in athletes and their attitudes toward psilocybin-assisted therapy in concussion recovery. Therapeutic Advances in Psychopharmacology 14, 20451253241264812. DOI: 10.1177/20451253241264812. 39132012 PMC11311162

[ref244] Vargas MV , Dunlap LE , Dong C , Carter SJ , Tombari RJ , Jami SA , Cameron LP , Patel SD , Hennessey JJ , Saeger HN , McCorvy JD , Gray JA , Tian L and Olson DE (2023) Psychedelics promote neuroplasticity through the activation of intracellular 5-HT2A receptors. Science 379(6633), 700–706. DOI: 10.1126/science.adf0435. 36795823 PMC10108900

[ref245] Vollenweider FX and Preller KH (2020) Psychedelic drugs: neurobiology and potential for treatment of psychiatric disorders. Nature Reviews Neurosciences 21(11), 611–624. DOI: 10.1038/s41583-020-0367-2. 32929261

[ref246] Wallach J , Cao AB , Calkins MM , Heim AJ , Lanham JK , Bonniwell EM , Hennessey JJ , Bock HA , Anderson EI , Sherwood AM , Morris H , de Klein R , Klein AK , Cuccurazzu B , Gamrat J , Fannana T , Zauhar R , Halberstadt AL and McCorvy JD (2023) Identification of 5-HT_2A_ receptor signaling pathways associated with psychedelic potential. Nature Communications 14(1), 8221. DOI: 10.1038/s41467-023-44016-1. PMC10724237 38102107

[ref247] Wang J , Xu D , Shen L , Zhou J , Lv X , Ma H , Li N , Wu Q and Duan J (2021a) Anti-inflammatory and analgesic actions of bufotenine through inhibiting lipid metabolism pathway. Biomedicine & Pharmacotherapy 140, 111749. DOI: 10.1016/j.biopha.2021.111749. 34058437

[ref248] Wang L , Wang Q , Wang W , Ge G , Xu N , Zheng D , Jiang S , Zhao G , Xu Y , Wang Y , Zhu R and Geng D (2021b) Harmine alleviates titanium particle-induced inflammatory bone destruction by immunomodulatory effect on the macrophage polarization and subsequent osteogenic differentiation. Frontiers in Immunology 12, 657687. DOI: 10.3389/fimmu.2021.657687. 34079546 PMC8165263

[ref249] Werle I and Bertoglio LJ (2024) Psychedelics: a review of their effects on recalled aversive memories and fear/anxiety expression in rodents. Neuroscience & Biobehavioral Reviews 167, 105899. DOI: 10.1016/j.neubiorev.2024.105899. 39305969

[ref250] Werle I , Nascimento LMM , Dos Santos ALA , Soares LA , Dos Santos RG , Hallak JEC and Bertoglio LJ (2024) Ayahuasca-enhanced extinction of fear behaviour: role of infralimbic cortex 5-HT_2A_ and 5-HT_1A_ receptors. British Journal of Pharmacology 181(11), 1671–1689. DOI: 10.1111/bph.16315. 38320596

[ref251] White SR , Obradovic T , Imel KM and Wheaton MJ (1996) The effects of methylenedioxymethamphetamine (MDMA, “Ecstasy”) on monoaminergic neurotransmission in the central nervous system. Progress in Neurobiology 49(5), 455–479. DOI: 10.1016/0301-0082(96)00027-5. 8895996

[ref253] Wittmann M , Carter O , Hasler F , Cahn BR , Grimberg U , Spring P , Hell D , Flohr H and Vollenweider FX (2007) Effects of psilocybin on time perception and temporal control of behaviour in humans. Journal of Psychopharmacology 21(1), 50–64. DOI: 10.1177/0269881106065859. 16714323

[ref254] Wolfson PE , Andries J , Feduccia AA , Jerome L , Wang JB , Williams E , Carlin SC , Sola E , Hamilton S , Yazar-Klosinski B , Emerson A , Mithoefer MC and Doblin R (2020) MDMA-assisted psychotherapy for treatment of anxiety and other psychological distress related to life-threatening illnesses: a randomized pilot study. Scientific Reports 10(1), 20442. DOI: 10.1038/s41598-020-75706-1. 33235285 PMC7686344

[ref255] Wsół A (2023) Cardiovascular safety of psychedelic medicine: current status and future directions. Pharmacological Reports 75(6), 1362–1380. DOI: 10.1007/s43440-023-00539-4. 37874530 PMC10661823

[ref256] Xin J , Ma X , Chen W , Zhou W , Dong H , Wang Z and Ji F (2021) Regulation of blood-brain barrier permeability by salvinorin a via alleviating endoplasmic reticulum stress in brain endothelial cell after ischemia stroke. Neurochemistry International 149, 105093. DOI: 10.1016/j.neuint.2021.105093. 34097989

[ref257] Yanakieva S , Polychroni N , Family N , Williams LTJ , Luke DP and Terhune DB (2019) The effects of microdose LSD on time perception: a randomized, double-blind, placebo-controlled trial. Psychopharmacology (Berl) 236(4), 1159–1170. DOI: 10.1007/s00213-018-5119-x. 30478716 PMC6591199

[ref258] Yao Y , Guo D , Lu TS , Liu FL , Huang SH , Diao MQ , Li SX , Zhang XJ , Kosten TR , Shi J , Bao YP , Lu L and Han Y (2024) Efficacy and safety of psychedelics for the treatment of mental disorders: a systematic review and meta-analysis. Psychiatry Research 335, 115886. DOI: 10.1016/j.psychres.2024.115886. 38574699

[ref259] Yerubandi A , Thomas JE , Bhuiya NMMA , Harrington C , Villa Zapata L and Caballero J (2024) Acute adverse effects of therapeutic doses of psilocybin: a systematic review and meta-analysis. JAMA Network Open 7(4), e245960. DOI: 10.1001/jamanetworkopen.2024.5960. 38598236 PMC11007582

[ref260] Yousefi P , Lietz MP , O’Higgins FJ , Rippe RCA , Hasler G , van Elk M and Enriquez-Geppert S (2025) Acute effects of psilocybin on attention and executive functioning in healthy volunteers: a systematic review and multilevel meta-analysis. Psychopharmacology (Berl). DOI: 10.1007/s00213-024-06742-2. PMC12084245 39847068

[ref261] Yu B , Becnel J , Zerfaoui M , Rohatgi R , Boulares AH and Nichols CD (2008) Serotonin 5-hydroxytryptamine(2A) receptor activation suppresses tumor necrosis factor-alpha-induced inflammation with extraordinary potency. Journal of Pharmacology and Experimental Therapeutics 327(2), 316–323. DOI: 10.1124/jpet.108.143461. 18708586

[ref262] Zanikov T , Gerasymchuk M , Ghasemi Gojani E , Robinson GI , Asghari S , Groves A , Haselhorst L , Nandakumar S , Stahl C , Cameron M , Li D , Rodriguez-Juarez R , Snelling A , Hudson D , Fiselier A , Kovalchuk O and Kovalchuk I (2023) The effect of combined treatment of psilocybin and eugenol on lipopolysaccharide-induced brain inflammation in mice. Molecules 28(6), 2624. DOI: 10.3390/molecules28062624. 36985596 PMC10056123

[ref263] Zaretsky TG , Jagodnik KM , Barsic R , Antonio JH , Bonanno PA , MacLeod C , Pierce C , Carney H , Morrison MT , Saylor C , Danias G , Lepow L and Yehuda R (2024) The psychedelic future of post-traumatic stress disorder treatment. Current Neuropharmacology 22(4), 636–735. DOI: 10.2174/1570159X22666231027111147. 38284341 PMC10845102

[ref264] Zeifman RJ , Singhal N , Dos Santos RG , Sanches RF , de Lima Osório F , Hallak JEC and Weissman CR (2021) Rapid and sustained decreases in suicidality following a single dose of ayahuasca among individuals with recurrent major depressive disorder: results from an open-label trial. Psychopharmacology (Berl) 238(2), 453–459. DOI: 10.1007/s00213-020-05692-9. 33118052

[ref265] Zeifman RJ , Wagner AC , Monson CM and Carhart-Harris RL (2023) How does psilocybin therapy work? an exploration of experiential avoidance as a putative mechanism of change. Journal of Affective Disorders Reports 334, 100–112. DOI: 10.1016/j.jad.2023.04.105. 37146908

[ref266] Zeng Y , Chourpiliadis C , Hammar N , Seitz C , Valdimarsdóttir UA , Fang F , Song H and Wei D (2024) Inflammatory biomarkers and risk of psychiatric disorders. JAMA Psychiatry 81(11), 1118–1129. DOI: 10.1001/jamapsychiatry.2024.2185. 39167384 PMC11339698

[ref267] Zheng ZH , Lin XC , Lu Y , Cao SR , Liu XK , Lin D , Yang FH , Zhang YB , Tu JL , Pan BX , Hu P and Zhang WH (2023) Harmine exerts anxiolytic effects by regulating neuroinflammation and neuronal plasticity in the basolateral amygdala. International Immunopharmacology 119, 110208. DOI: 10.1016/j.intimp.2023.110208. 37150016

[ref268] Zhong Z , Tao Y and Yang H (2015) Treatment with harmine ameliorates functional impairment and neuronal death following traumatic brain injury. Molecular Medicine Reports 12(6), 7985–7991. DOI: 10.3892/mmr.2015.4437. 26496827 PMC4758275

